# Identification and Analysis of the Paulomycin Biosynthetic Gene Cluster and Titer Improvement of the Paulomycins in *Streptomyces paulus* NRRL 8115

**DOI:** 10.1371/journal.pone.0120542

**Published:** 2015-03-30

**Authors:** Jine Li, Zhoujie Xie, Min Wang, Guomin Ai, Yihua Chen

**Affiliations:** State Key Laboratory of Microbial Resources, Institute of Microbiology, Chinese Academy of Sciences (CAS), Beijing, 100101, P. R. China; University Paris South, FRANCE

## Abstract

The paulomycins are a group of glycosylated compounds featuring a unique paulic acid moiety. To locate their biosynthetic gene clusters, the genomes of two paulomycin producers, *Streptomyces paulus* NRRL 8115 and *Streptomyces sp*. YN86, were sequenced. The paulomycin biosynthetic gene clusters were defined by comparative analyses of the two genomes together with the genome of the third paulomycin producer *Streptomyces albus* J1074. Subsequently, the identity of the paulomycin biosynthetic gene cluster was confirmed by inactivation of two genes involved in biosynthesis of the paulomycose branched chain (*pau11*) and the ring A moiety (*pau18*) in *Streptomyces paulus* NRRL 8115. After determining the gene cluster boundaries, a convergent biosynthetic model was proposed for paulomycin based on the deduced functions of the *pau* genes. Finally, a paulomycin high-producing strain was constructed by expressing an activator-encoding gene (*pau13*) in *S*. *paulus*, setting the stage for future investigations.

## Introduction

Natural products from *Streptomyces* are a prolific source of therapeutic agents. Many of them have already been used clinically as antibiotics, immunosuppressants and antitumor drugs. Typically, the structure, regulatory and resistance genes of those compounds are clustered in the genomes, which significantly facilitates biosynthetic investigations [1]. Over the past three decades, the biosynthetic and regulatory mechanisms of a great number of *Streptomyces* natural products have been deciphered, which enables us to generate novel compounds through combinatorial biosynthesis or synthetic biology approaches and to improve their titers in a strategic manner [2, 3].

**Fig 1:**
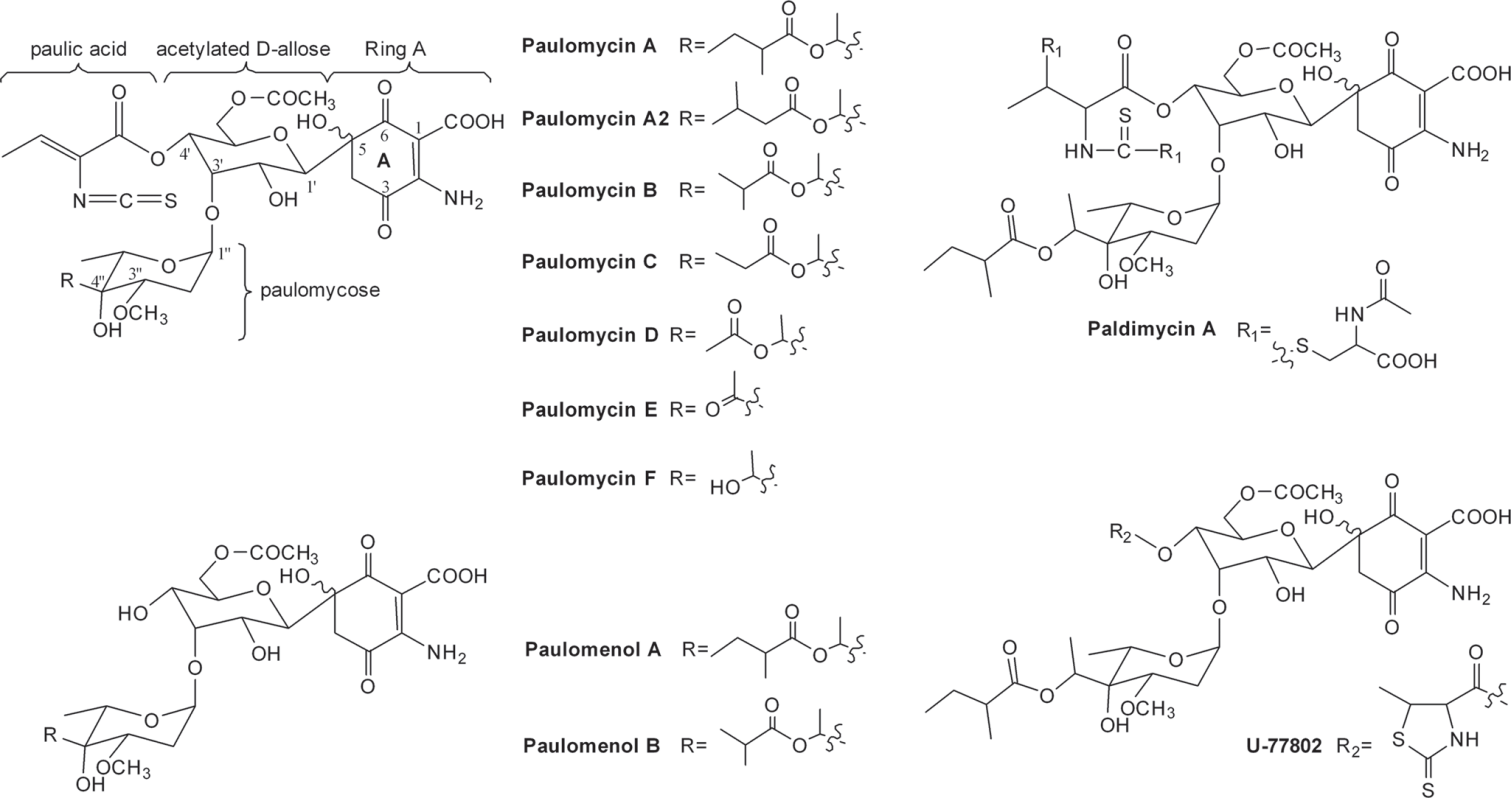
Structures of the paulomycins, paulomenols and two of their analogs decorated at the paulic acid moiety, paldimycin A and U-77802.

The paulomycins are a group of glycosylated compounds isolated from several different *Streptomyces* strains (Fig. 1). Before Argoudelis *et al*. solved the structures of paulomycin A and B from *Streptomyces paulus sp*. 273 and named them in 1982 [4], these compounds were reported using various names, such as U-43120 [5], NSC-163500 [6] and volonomycins [7]. Paulomycin A is composed of a quinone-like ring A, an acetylated D-allose, an unusual eight-carbon sugar paulomycose esterified by 2-methylbutyric acid at the branched hydroxyl group, and a unique paulic acid moiety containing a rare isothiocyanate group. Unlike paulomycin A, the paulomycose branched hydroxyl group is decorated by isobutyric acid in paulomycin B. A series of paulomycins with various modifications at the two-carbon branched chain of paulomycose were isolated subsequently from several *S*. *paulus* strains and *Streptomyces albus* J1074 (Fig. 1) [8–10]. In addition, there are some paulomycin analogs modified at the paulomycose 3″-methoxyl group, such as O-demethylpaulomycins (lack the 3″-O-methyl group) [11] and senfolomycins. Although the senfolomycins were discovered much earlier than paulomycins [12], their structures were not determined until 1988. Senfolomycin A and B are identical to paulomycin E and F respectively, but possess reversed configuration at the 3″-methoxyl groups [13]. This structural diversity is further expanded by a variety of paulomycin analogs such as the paulomenols (lacking the paulic acid moiety) U-77802 and U-77803 (the hydrogen sulfide adducts of paulomycin A and B) and the paldimycins (with N-acetyl-L-cysteine attached at the isothiocyanate moiety) [14] (Fig. 1). All paulomycins and their analogs have shown excellent antibiotic activity against Gram-positive bacteria [11, 15, 16], and some exhibit substantial activity against a range of other microorganisms that may be useful for treating urethritis and *Chlamydia* infections [17].

To our knowledge, all known paulomycin compounds have only been produced by *Streptomyces*, and no chemical synthesis has been reported. However, the limited understanding of paulomycin biosynthesis currently impedes the expansion of structural diversity through combinatorial biosynthesis. We were therefore motivated to define the paulomycin biosynthetic gene cluster by sequencing the genomes of two paulomycin producers, *S*. *paulus* NRRL 8115 [6] and *Streptomyces sp*. YN86 [18]. The paulomycin gene clusters were localized by comparative genomic analyses of the two strains and another paulomycin producer *S*. *albus* J1074 [10]. After determining the paulomycin gene cluster boundaries in *S*. *paulus* NRRL 8115, we proposed a putative paulomycin biosynthetic pathway based on bioinformatic analysis. Finally, production of the paulomycins in *S*. *paulus* NRRL 8115 was improved considerably by overexpressing an activator encoding gene *pau13*.

## Materials and Methods

### Bacterial strains, media and plasmids

The strains and plasmids used in this study are summarized in S1 Table. *Escherichia coli* JM109 was used as a host to prepare plasmids. *E*. *coli* ET12567/pUZ8002 was used for *E*. *coli*-*Streptomyces* conjugation [19]. All *E*. *coli* strains were incubated in Luria-Bertani medium at 37°C. *Streptomyces paulus* NRRL8115, *Streptomyces sp*. YN86 and *Streptomyces albus* J1074 were the three paulomycin producers. For spore formation, *Streptomyces* strains were grown on mannitol/soya (MS) agar at 28°C [19]. Liquid YEME medium was used for *Streptomyces* genomic DNA isolation [19]. Medium GS-7 [7] was used as the seed culture and medium R5α was used as the paulomycin production medium for *S*. *paulus* NRRL8115 [20]. Plasmids pUC119::KanR, pKC1132 and pSET152::ermE* were described previously [19, 21, 22].

### DNA manipulation

Isolation of *Streptomyces* genomic DNAs was performed as previously described [19]. PCRs were performed with Taq DNA polymerase (TransGene, Beijing, China) or KOD-Plus DNA polymerase (Toyobo, Osaka, Japan) according to the manufacturer’s instructions. Restriction enzyme digestions, ligations and transformations were performed following the standard methods [23]. *E*. *coli-Streptomyces* conjugations were carried out following the described protocols [19].

### Sequencing and bioinformatics analyses

Genomic DNA sequencing service was provided by Majorbio Company (Majorbio, Shanghai, China) using Illumina Hiseq2000 system. Analyses of the secondary metabolite gene clusters were performed with antiSMASH (http://antismash.secondarymetabolites.org/) [24]. The possible open reading frames (ORFs) were predicted by Prodigal (http://prodigal.ornl.gov/) [25]. The gene functional annotations combined the search results of NCBI and KEGG databases. Multiple alignments were performed with CLUSTALW. The sequences of the paulomycin biosynthetic gene clusters from *S*. *paulus* NRRL8115 and *S*. *sp*. YN86 have already been submitted to GenBank (accession number KJ721164 and KJ721165).

### Production of paulomycin

For paulomycin production, 50 μL *S*. *paulus* NRRL8115 spores were inoculated into GS-7 medium and cultured at 28°C for 2 days. The resulting seed culture was inoculated into 50 mL medium R5α at 2% ratio (v/v) and cultured for 4 days. The fermentation broth was harvested by centrifugation and extracted with 50 mL ethyl acetate for three times. The ethyl acetate extraction was dried in *vacuo*. It was then redissolved in 1 mL acetonitrile and subjected to HPLC analysis.

### Construction of mutants CIM3001 (*S*. *paulus pau11*::*aph*) and CIM3002 (*S*. *paulus pau18*::*aph*)

An allelic replacement strategy was used to inactivate the target genes individually in *S*. *paulus* NRRL 8115. The 2.4-kb fragment upstream of *pau11* was PCR amplified with primer pair pau11-up-F and pau11-up-R and inserted into the *Pst*I/*Xba*I sites of pUC119::KanR to construct pCIM3001 (all primers used in this study are listed in S2 Table. The restriction site used in plasmid construction is underlined and marked after each primer). The 1.1-kb fragment downstream of *pau11* was obtained by PCR using primer pair pau11-down-F and pau11-down-R and cloned into the *Eco*RI site of pCIM3001 via a ligation-independent cloning strategy [26] to generate pCIM3002. The fidelity of all the PCR cloned fragments was confirmed by sequencing. The 4.5-kb mutant allele containing the two fragments flanking *pau11* and the kanamycin resistance gene (*aph*) was excised by *Pst*I/*Eco*RI and inserted into the same sites of pKC1132 to construct pCIM3003. Plasmid pCIM3003 was then introduced into *S*. *paulus* NRRL 8115 via *E*. *coli-Streptomyces* conjugation. Exconjugants with kanamycin resistance and apramycin sensitivity were selected as the desired *S*. *paulus pau11*::*aph* mutant strain CIM3001. The genotype of CIM3001 was confirmed by PCR with primers pau11-up-F and pau11-down-R. Subsequent *Xba*I digestions of the PCR products were carried out to clearly discriminate between mutants and wild-type (S1 Fig.).

To inactivate the *pau18* gene, the 0.7-kb upstream fragment was PCR cloned with primer pair pau18-up-F and pau18-up-R, and the 1.3-kb downstream fragment was cloned with primer pair pau18-down-F and pau18-down-R. The two fragments were inserted into the *Pst*I/*Bam*HI and *Kpn*I/*Eco*RI sites of pUC119::KanR sequentially to generate pCIM3004. The 3.9-kb mutant allele containing the two fragments flanking *pau18* and the kanamycin resistance cassette was then cut off from pCIM3004 by *Pst*I/*Eco*RI and inserted into the same sites of pKC1132 to construct pCIM3005. After introducing plasmid pCIM3005 into *S*. *paulus* NRRL 8115 via *E*. *coli-Streptomyces* conjugation, exconjugants that were kanamycin resistant and apramycin sensitive were selected as the desired *S*. *paulus pau18*::*aph* mutant strain CIM3002. The genotype of CIM3002 was confirmed by PCR with primers pau18-up-F and pau18-down-R (S1 Fig.).

### Complementation of CIM3001 and CIM3002

The 1.0-kb fragment containing the whole *pau11* gene was PCR-amplified from *S*. *paulus* NRRL 8115 with primer pair pau11-E-F and pau11-E-R and inserted into the *Nde*I/*Bam*HI sites of pSET152::ermE* to generate plasmid pCIM3006. Introduction of pCIM3006 into *S*. *paulus* CIM3001 generated the *S*. *paulus pau11*::*aph* complemented strain CIM3003.

Similarly, the 2.0-kb fragment harboring the whole *pau18* gene was cloned by PCR with primer pair pau18-E-F and pau18-E-R and inserted into the *Nde*I/*Bam*HI sites of pSET152::ermE* to generate pCIM3007. The *S*. *paulus pau18*::*aph* complemented strain CIM3004 was obtained by introduction of pCIM3007 into *S*. *paulus* CIM3002.

### Construction of the mutants for determination of the paulomycin gene cluster boundaries

Details are described in S1 File. The genotype of the *S*. *paulus* mutants were verified by PCR (S2–S3 Figs.).

### Construction and complementation of the CIM3005 (*S*. *paulus pau13*::*aph*) mutant strain

The two fragments flanking *pau13* were amplified by PCR using primer pair pau13-up-F and pau13-up-R for the 1.1-kb upstream fragment and primer pair pau13-down-F and pau13-down-R for the 1.8-kb downstream fragment. The two fragments were cloned into the *Eco*RI/*Kpn*I and *BamH*I/*Pst*I sites of pUC119::KanR sequentially to generate pCIM3008. The 3.9-kb mutant allele containing both the up- and down-stream fragments and the kanamycin resistance cassette was then excised by *Eco*RI/*Pst*I and inserted into the same sites of pKC1132 to generate pCIM3009. Plasmid pCIM3009 was introduced into *S*. *paulus* NRRL 8115 via *E*. *coli-Streptomyces* conjugation. Exconjugants that were kanamycin resistant and apramycin sensitive were picked out as the *S*. *paulus pau13*::*aph* mutant strain CIM3005, the genotype of which was then confirmed by PCR with primers pau13-up-F and pau13-down-R and subsequent *Bam*HI digestions of the PCR products (S1 Fig.).

The 1.0-kb fragment containing the whole *pau13* gene was amplified by PCR using primers pau13-E-F and pau13-E-R and inserted into the same sites of pSET152::ermE* to construct pCIM3010. The *pau13* mutant complemented strain CIM1006 was constructed by introduction of pCIM3010 into CIM3005 via *E*. *coli-Streptomyces* conjugation.

### Overexpression of *pau13* in *S*. *paulus* NRRL8115

Introduction of pCIM3010 into *S*. *paulus* NRRL 8115 via *E*. *coli-Streptomyces* conjugation generated the recombination strain CIM3007, in which the expression of *pau13* is under the control of the constitutive ermE* promoter. For comparisons of the paulomycin and paulomenol titers in wild-type and CIM3007 strains, standard error values are obtained from at least three independent cultures.

### Analytical and Spectroscopic Procedures

HPLC analyses were carried out with an Apollo C18 column (5 μm, 4.6 × 250mm, Alltech, Deerfield, IL, USA) with Shimadzu HPLC system (Shimadzu, Kyoto, Japan). The column was developed with a linear gradient using acetonitrile and water with 0.1% trifluoroacetic acid at a flow rate of 0.8 mL/min. For the first 5 minutes, the ratio of acetonitrile was maintained at 5%, and it was changed linearly from 5% to 90% over 5–25 min and from 90% to 100% over 25–30 min. The detection wavelength was 320 nm.

MS and tandem MS were performed on an Agilent 1260/6460 Triple-Quadrupole LC/MS system (Agilent, Santa Clara, CA, USA) with the electrospray ionization source. The high resolution MS analysis was performed on an Aligent 1200 HPLC system and 6520 QTF-MS system (Agilent, Santa Clara, CA, USA). The mass spectrometer scanned from *m/z* = 100–1500 in negative ion mode.

## Results and Discussion

### Comparative genomic analyses to define the paulomycin biosynthetic gene cluster

**Fig 2:**
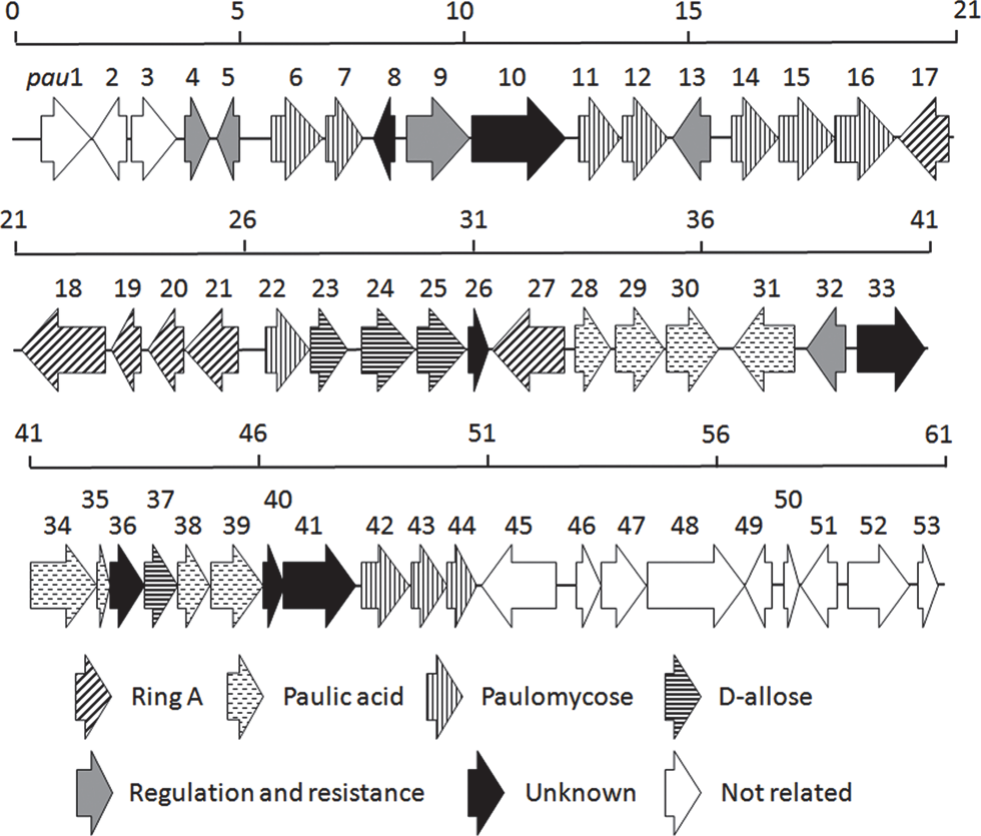
Genetic organization of the *pau* gene cluster from *S*. *paulus* NRRL 8115. Proposed functions for individual ORFs are coded with various patterns and summarized in Table 1.

Three *Streptomyces* strains (*S*. *paulus* NRRL 8115, *S*. *albus* J1074 and *S*. *sp*. YN86) were used for searching the paulomycin biosynthetic gene clusters. The genome sequence of *S*. *albus* J1074 is available in GenBank (accession No. NC_020990). By draft sequencing the genomes of *S*. *paulus* NRRL 8115 and *S*. *sp*. YN86, we now have three sequenced genomes harboring the paulomycin gene cluster. Since paulomycin contains two sugars attached by a C- or O-glycosidic bond, we searched by comparative genomic analysis and identified several conserved gene clusters containing glycosyltransferase-encoding genes in the three genomes as candidates. Furthermore, considering that paulomycin contains the eight-carbon sugar paulomycose with a two-carbon branched chain, we searched these clusters for the presence of genes involved in the two-carbon branched chain biosynthesis of high-carbon-chain sugars. To our knowledge, only two molecular mechanisms have been described for the formation of sugar’s two-carbon branched chain. One is catalyzed by the pyruvate dehydrogenase-like proteins (AviB1/AviB2) and the nonheme iron-dependent enzyme AviO2 involved in the methyleurekanate biosynthesis of avilamycin from *Streptomyces viridochromogenes* Tü57 [27]; the other is the TPP-dependent flavoprotein YerE involved in yersiniose biosynthesis from *Yersina pseudotuberculosis* [28]. Further bioinformatic analysis revealed a 61-kb DNA region containing both the AviB1/AviB2 homologs and the glycosyltransferase-encoding genes in each of the three strains, which is regarded as the putative paulomycin biosynthetic gene cluster. The 61-kb region is highly conserved in both gene organization and individual gene functions among all three paulomycin producers and the paulomycin biosynthetic gene cluster from *S*. *paulus* NRRL 8115 (named as the *pau* gene cluster) is depicted in Fig. 2. Functions of the 53 open reading frames (ORFs) were assigned by careful bioinformatic analyses (Table 1).

**Table 1:** Homologous proteins of ORFs in the paulomycin biosynthetic gene clusters.

Genes	Sizes ^[^ ^a^ ^]^	Proposed Functions	Protein Homologs ^[^ ^b^ ^]^(identity/similarity)	Protein Homologs in *S*. *sp*. YN86 ^[^ ^c^ ^]^	Protein Homologs in *S*. *albus* J1074 ^[^ ^c^ ^]^
*pau1*	382	RNA polymerase sigma factor	(NP_628619, 82/89)	PauY1 (98/99)	YP_007744020 (98/99)
*pau2*	267	M23 family peptidase	(WP_018522979, 82/87)	PauY2 (100/100)	YP_007744019 (99/99)
*pau3*	337	aminotransferase	(WP_019061967, 63/69)	PauY3 (90/90)	YP_007744018 (91/91)
*pau4*	187	TetR-family transcriptional regulator	(NP_630249, 59/76)	PauY4 (96/97)	YP_007744017 (96/97)
*pau5*	173	LuxR family regulator	(WP_005479035, 51/69)	PauY5 (52/52)	YP_007744016 (50/50)
*pau6*	389	isovaleryltransferase	MppM (AAU34206, 37/52) [31]	PauY6 (99/99)	YP_007744015 (100/100)
*pau7*	272	Aldo-keto reductase	(NP_630632, 64/77)	PauY7 (98/99)	YP_007744014 (100/100)
*pau8*	162	hypothetical protein	(NP_625096, 55/69)	PauY8 (100/100)	YP_007744013 (100/100)
*pau9*	480	EmrB/QacA subfamily transporter	AsuM1 (ADI58655, 35/54) [48]	PauY9 (100/100)	YP_007744012 (100/100)
*pau10*	709	elongation factor G	(NP_628821, 90/95)	PauY10 (100/100)	YP_007744011 (100/100)
*pau11*	325	pyruvate dehydrogenase α subunit	AviB1 (AAK83190, 37/47) [27]	PauY11 (100/100)	YP_007744010 (100/100)
*pau12*	345	pyruvate dehydrogenase β subunit	AviB2 (AAK83191, 34/45) [27]	PauY12 (100/100)	YP_007744009 (99/100)
*pau13*	286	SARP family transcriptional regulator	SrrZ (WP_023415585, 51/65) [49]	PauY13 (100/100)	YP_007744008 (100/100)
*pau14*	361	glycosyltransferase auxiliary protein	DesVIII (AAC68676, 28/37) [46]	PauY14 (98/98)	YP_007744007 (99/99)
*pau15*	426	O-glycosyltransferase	DesVII (AAC68677, 43/58) [46]	PauY15 (99/100)	YP_007744006 (99/100)
*pau16*	461	NDP-hexose 2,3-dehydratase	SnogH (CAA12009, 54/64) [50]	PauY16 (99/99)	YP_007744005 (100/100)
*pau17*	389	3-hydroxybenzoate 6-hydroxylase	XlnD (Q9F131, 44/60) [51]	PauY17 (100/100)	YP_007744004 (99/100)
*pau18*	651	phenazine biosynthesis protein PhzE	PhzE (NP_250594, 53/65) [33]	PauY18 (100/100)	YP_007744003 (100/100)
*pau19*	219	phenazine biosynthesis protein PhzD	PhzD (NP_250593, 56/70) [33]	PauY19 (99/99)	YP_007744002 (99/99)
*pau20*	261	2, 3-dihydro-2, 3-dihydroxybenzoate dehydrogenase	MxcC (AAG31126, 58/67) [52]	PauY20 (100/100)	YP_007744001 (99/99)
*pau21*	402	3-deoxy-D-arabino-heptulosonate-7-phosphate synthase	PlmI (AAQ84163, 50/60) [53]	PauY21 (100/100)	YP_007744000 (99/100)
*pau22*	331	dTDP-glucose 4,6-dehydratase	CpzDIII (ADI50277, 65/76) [54]	PauY22 (100/100)	YP_007743999 (100/100)
*pau23*	289	hexose-1-phosphate thymidylyltransferase	DesIII (AAC68682, 59/73) [55]	PauY23 (100/100)	YP_007743998 (99/99)
*pau24*	404	acyltransferase	MppN (AAU34207, 36/50) [31]	PauY24 (100/100)	YP_007743997 (100/100)
*pau25*	375	C-glycosyltransferase	SaqGT5 (ACP19370, 50/64) [56]	PauY25 (99/100)	YP_007743996 (100/100)
*pau26*	145	glyoxalase	(WP_005631538, 29/38) [57]	PauY26 (100/100)	-^[^ ^d^ ^]^
*pau27*	547	FAD-binding monooxygenase	TcmG (AAA67511, 37/49) [58]	PauY27 (100/100)	YP_007743995 (100/100)
*pau28*	255	reductase	AntM (AGG37759, 48/64) [59]	PauY28 (98/99)	YP_007743994 100/100)
*pau29*	349	3-oxoacyl-ACP synthase III	CosE (ABC00733, 43/58) [35]	PauY29 (100/100)	YP_007743993 (100/100)
*pau30*	380	UBA/THIF-type NAD/FAD binding protein	MoeZ (CAB08310, 40/58) [42]	PauY30 (99/99)	YP_007743992 (100/100)
*pau31*	455	cysteine desulfurase	SufS (AHG14743, 27/43) [41]	PauY31 (99/99)	YP_007743991 (99/100)
*pau32*	290	putative regulatory protein	PhpR (AAU00093, 23/36)	PauY32 (100/100)	YP_007743990 (100/100)
*pau33*	496	oxidoreductase	KedU31 (AFV52193, 26/38) [60]	PauY33 (99/99)	YP_007743989 (100/100)
*pau34*	478	acyl-CoA synthase	(AFV52195, 43/57)	PauY34 (100/100)	YP_007743988 (100/100)
*pau35*	98	acyl-carrier protein	KedU34 (AFV52196, 24/43) [60]	PauY35 (100/100)	YP_007743987 (100/100)
*pau36*	250	dihydrodipicolinate reductase	DapB (AAW38176, 18/32) [61]	PauY36 (100/100)	YP_007743986 (100/100)
*pau37*	249	ribulose-5-phosphate 4-epimerase	AraD (NP_414603, 19/30) [30]	PauY37 (99/100)	YP_007743985 (99/100)
*pau38*	228	4'-phosphopantetheinyl transferase	AlpN (AAR30158, 19/30) [62]	PauY38 (99/99)	YP_007743984 (99/99)
*pau39*	380	acyl-CoA dehydrogenase domain-containing protein	AcdH (AAD44196, 32/49) [63]	PauY39 (100/100)	YP_007743983 (100/100)
*pau40*	156	hypothetical protein	SAV_915 (NP_822090, 28/39)	PauY40 (99/99)	YP_007743982 (99/99)
*pau41*	527	pyranose oxidase	CetL (ACH85575, 25/33) [64]	PauY41 (100/100)	YP_007743981 (100/100)
*pau42*	345	TDP-4-keto-6-deoxyhexose 2, 3-reductase	LipDig3 (ABB05109, 62/73) [65]	PauY42 (99/99)	YP_007743980 (100/100)
*pau43*	257	TDP-6-deoxy-L-hexose 3-O-methyltransferase	CalS11 (4GF5_A, 64/74) [66]	PauY43 (100/100)	YP_007743979 (100/100)
*pau44*	220	TDP-4-keto-6-deoxyhexose 3, 5-epimerase	AveBV (NP_822124, 59/65) [45]	PauY44 (100/100)	YP_007743978 (100/100)
*pau45*	546	malate synthase	AceB (AAG29597, 85/91)	PauY45 (100/100)	YP_007743977 (100/100)
*pau46*	181	xanthine dehydrogenase, 2Fe-2S subunit	YagT (YP_488582, 43/53)	PauY46 (99/99)	YP_007743976 (98/98)
*pau47*	328	oxidoreductase with FAD-binding domain	YagS (YP_488581, 52/63)	PauY47 (99/99)	YP_007743975 (100/100)
*pau48*	703	oxidoreductase with molybdenum-binding domain	YagR (YP_488580, 37/51)	PauY48 (100/100)	YP_007743974 (100/100)
*pau49*	194	YgfJ family molybdenum hydroxylase accessory protein	MobA (NP_418294, 23/34)	PauY49 (98/99)	YP_007743973 (99/99)
*pau50*	111	hypothetical protein	(NP_630345, 48/59)	PauY50 (98/100)	YP_007743972 (97/99)
*pau51*	268	IclR family transcriptional regulator	(NP_630346, 83/89)	PauY51 (100/100)	YP_007743971 (100/100)
*pau52*	444	allantoinase	AllB (NP_630347, 76/85)	PauY52 (99/100)	YP_007743970 (100/100)
*pau53*	142	guanyl-specific ribonuclease	LipX1 (ABB05092, 63/72)	PauY53 (99/99)	YP_007743969 (99/99)

[a] Numbers are in amino acids.

[b] Given in brackets are GenBank accession numbers and percentage identity/percentage positive. References are added in case of available.

[c] Given in brackets is percentage identity/percentage positive.

[d] Not exist in *S*. *albus* J1074.

### Confirmation of the *pau* gene cluster in *S*. *paulus* NRRL 8115

During our study, it was observed that production of the paulomycins in *S*. *albus* J1074 is not stable. Given that the genetic manipulation in *S*. *paulus* NRRL 8115 is much easier than that in *S*. *sp*. YN86, *S*. *paulus* NRRL 8115 was used as a model system in the following paulomycin biosynthetic studies. The identities of paulomycin A, paulomycin B, paulomenol A and paulomenol B produced by *S*. *paulus* NRRL 8115 were confirmed by ultraviolet-visible absorption spectra, high resolution mass spectrometry (MS) and tandem MS (S4–S7 Figs.).

**Fig 3:**
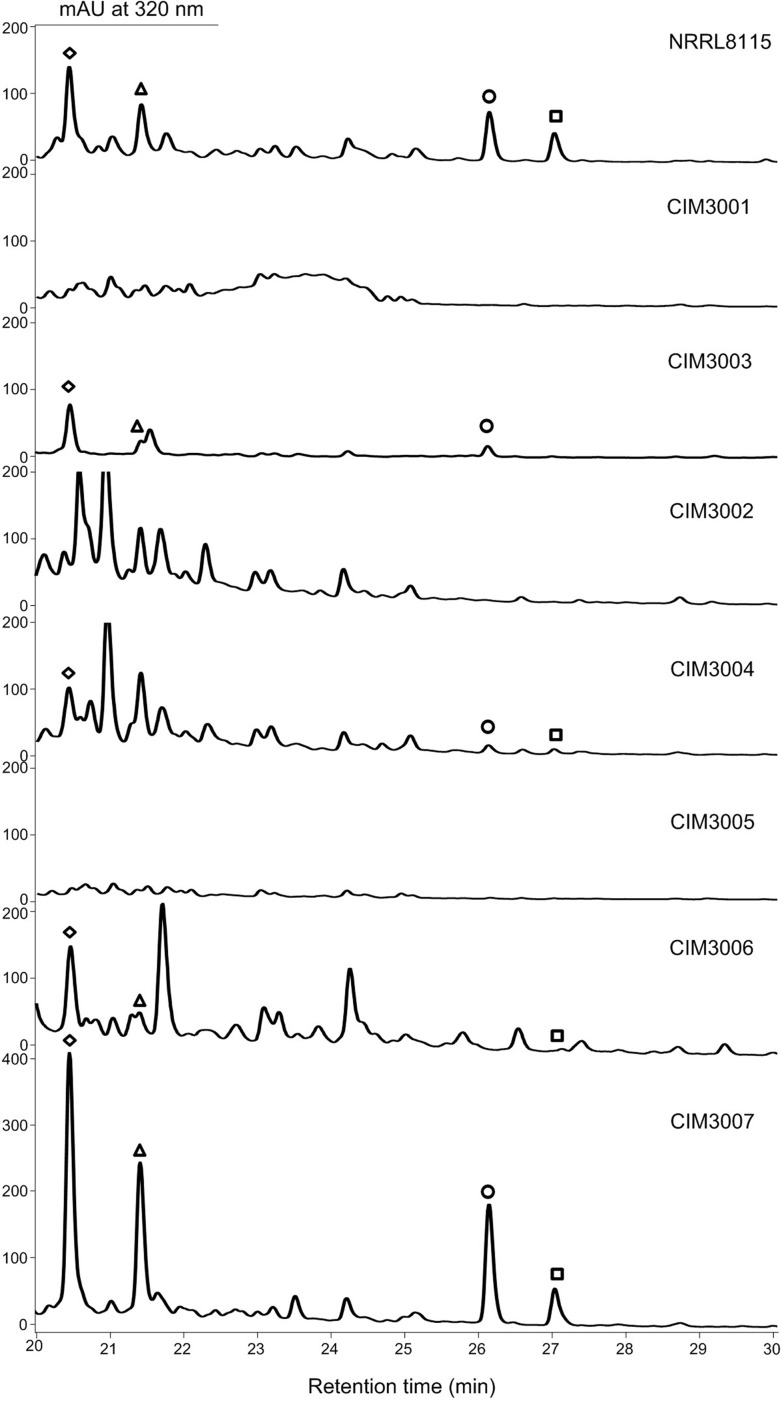
HPLC traces of selected *S*. *paulus* mutants and recombinant strains. CIM3001, the *pau11* inactivated mutant; CIM3003, complemented strain of CIM3001; CIM3002, the *pau18* inactivated mutant; CIM3004, complemented strain of CIM3002; CIM3005, the *pau13* inactivated mutant; CIM3006, complemented strain of CIM3005; CIM3007, *S*. *paulus* recombinant strain overexpressing the *pau13* gene. Paulomycin A (□); Paulomycin B (○); Paulomenol A (⋄); Paulomenol B (Δ).

To obtain direct proof that the *pau* cluster is crucial for the production of paulomycins in *S*. *paulus* NRRL 8115, two genes in the cluster (*pau11* and *pau18*) were mutated individually by targeted gene replacement (S1 Fig.). The gene product of *pau11* is an AviB1 homologue involved in the eight-carbon sugar paulomycose biosynthesis; the gene product of *pau18* is a putative 2-amino-2-deoxyisochorismate (ADIC) synthase responsible for the biosynthesis of the ring A moiety (see the following text). The two *S*. *paulus* mutants CIM3001 (*S*. *paulus pau11*::*aph*), and CIM3002 (*S*. *paulus pau18*::*aph*) were then cultured in the same condition as that for paulomycin production in the wild-type strain and checked by HPLC (Fig. 3). The production of paulomycins (A and B) and paulomenols (A and B) was totally abolished in the two mutants confirming that the *pau* cluster is involved in paulomycin biosynthesis. Recent mining of the *S*. *albus* J1074 genome identified the same region for paulomycin biosynthesis by comparing the metabolic profiles of the wild-type strain and a mutant control with two genes *sshg_05327* (encoding Pau18 homolog) and *sshg_05328* (encoding Pau19 homolog) deleted [29]. Complementation with constitutively expressed *pau11* and *pau18* genes in trans restored production of paulomycins and paulomenols in the two complemented strains CIM3003 and CIM3004, respectively, excluding a possible polar effect.

### Determination of the *pau* cluster boundaries

To determine the paulomycin biosynthetic gene cluster boundaries, selective ORFs at both ends of the *pau* cluster were inactivated systematically by targeted gene replacement (S2–S3 Figs.). Inactivation of *pau7* and *pau43* dramatically reduced or blocked the production of the paulomycins and the paulomenols, implying that these genes are involved in paulomycin biosynthesis. In contrast, inactivation of *pau1*, *pau3*, *pau45*, *pau48* and *pau52* did not diminish the production of paulomycin analogs (S8 Fig.), suggesting they are outside of the *pau* gene cluster. Consequently, the *pau* gene cluster was narrowed down to a 48-kb region of DNA containing 41 ORFs, from *pau4* to *pau44*, based on the gene inactivation data and the predicted functions of the *pau* genes.

### Biosynthesis of the D-allose moiety

**Fig 4:**
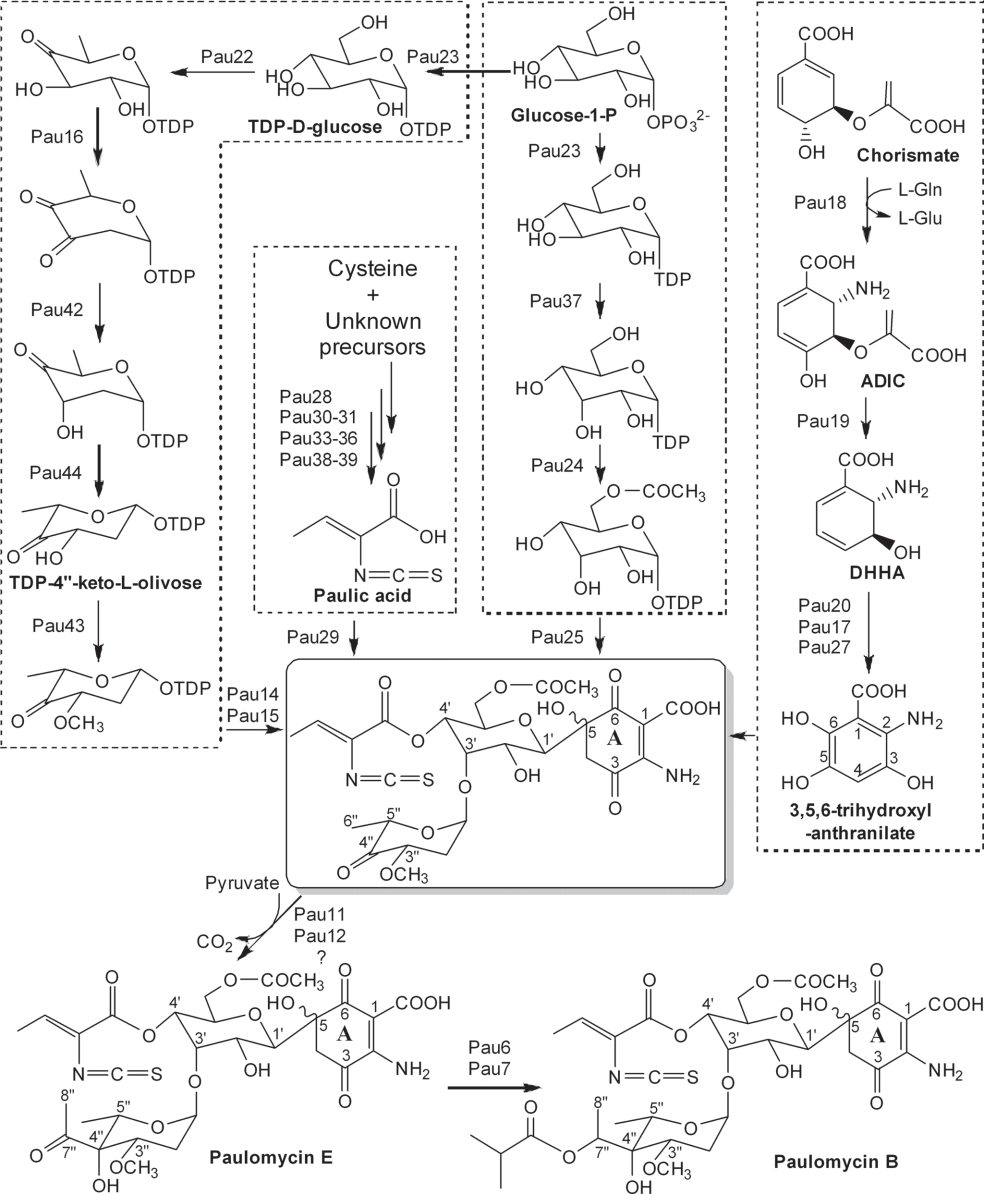
A proposed convergent model of paulomycin biosynthesis.

A convergent model of paulomycin biosynthesis was proposed on the basis of the deduced functions of the genes within the cluster (Fig. 4). Four genes (*pau23-pau25* and *pau37*) in the *pau* cluster are suggested to be responsible for the D-allose moiety biosynthesis. A plausible biosynthetic pathway is that Pau23, a putative hexose-1-phosphate thymidylyltransferase, activates the D-glucose-1-phosphate to TDP-D-glucose, which was then epimerized by Pau37, a homolog of ribulose-5-phosphate 4-epimerase AraD fr*om Escherichia coli* [30], to form TDP-D-allose. Subsequently, the C-glycosyltransferase homolog Pau25 transfers the activated D-allose to the paulomycin ring A at the C-5 position. Pau24 is a homolog of the acyltransferase MppN from *Streptomyces hygroscopicus* NRRL 30439 and is suggested to be responsible for addition of the acetyl group to the 6ˊ-OH of D-allose [31].

### Biosynthesis of the ring A moiety

It is proposed that the quinone-like ring A is derived from chorismate; six genes (*pau17*-*pau21* and *pau27*) are assumed to be involved in the biosynthesis of this moiety. Pau21 is a 3-deoxy-D-arabino-heptulosonate-7-phosphate synthase responsible for condensation of phosphoenolpyruvate and erythrose 4-phosphate to 2-keto-3-deoxy-D-arabinoheptulosonate-7-phosphate, the first intermediate of the shikimate pathway [32], which is then converted to chorismate by a number of enzymes from the primary metabolic pathway. Chorismate is the branch point of the shikimate pathway, with one branch leading to phenazines through 2-amino-2-deoxyisochorismate (ADIC) and *trans*-2,3-dihydro-3-hydroxyanthranilic acid (DHHA) by ADIC synthase and isochorismatase [33, 34]. The early steps of the paulomycin ring A biosynthesis should follow an analogous route that includes a same set of transformations to generate DHHA by Pau18 (ADIC synthase) and Pau19 (isochorismatase). DHHA is further converted to 3,5,6-trihydroxyl-anthranilate by the dehydrogenase Pau20 (aromatization) and the two monooxygenases Pau17 and Pau27 (C-5 and C-6 hydroxylations) in an order yet to be determined.

### Biosynthesis of the paulic acid

The paulic acid is unique and not much is known about its biosynthesis. The lack of an acyl-CoA ligase-encoding gene in the *pau* gene cluster indicates an unusual mechanism for the paulic acid installation. Our bioinformatic analysis of Pau29 suggests it is a ketoacylsynthase III-like acyltransferase catalyzing ester bond formation between paulic acid and the D-allose moiety. Pau29 shows 43% identity to CosE from *Streptomyces olindensis* [35] and 33% identity to CerJ from *Streptomyces tendae* [36]. CosE is a ketoacylsynthase III type condensation enzyme responsible for the propionyl-CoA starter unit loading in cosmomycin biosynthesis [35]; CerJ is a ketoacylsynthase III-like acyltransferase appending a dimethylmalonyl moiety to the hydroxyl group of the cervimycin sugar via ester bond formation [36]. A unifying feature of the CosE homologs is that they possess a highly conserved catalytic triad Cys(Ser)-His-His, in which the first residue (Cys or Ser) is essential for transacylation and the other two His residues are indispensible for the decarboxylative condensation. In CerJ, a Val substitution in the first conserved His in that catalytic triad is consistent with its loss of condensation function. Notably CerJ has a new Cys-His-Asp catalytic triad that is crucial for its acyltransferase activity [36]. Careful analysis of Pau29 revealed that it also lacks the first conserved His essential for the decarboxylative condensation in CosE homologs and features a Ser-His-Asp motif similar to CerJ (S9 Fig.), further supporting its proposed role as an acyltransferase. In addition to the aforementioned CerJ, there are several examples of condensation-like enzymes catalyzing ester bond formation including XclB/XclC [37], NonJ/NonK [38] and SgcC5 [39] involved in xenocyloin, nonactin and C-1027 biosynthesis, respectively. They all use CoA, or carrier protein-tethered fatty acids, or amino acids as substrates, indicating that Pau29 requires a CoA or ACP tethered paulic acid (or an acid intermediate) as a substrate, which may plausibly be synthesized from unknown precursors by related enzymes such as Pau28 (a putative oxidoreductase catalyzing the acyl carrier protein (ACP)-tethered ketoreduction), Pau34 (acyl-CoA synthase), Pau35 (ACP), Pau38 (phosphopantetheinyl transferase) and Pau39 (acyl-CoA dehydrogenase).

For the biosynthesis of the unusual isothiocyanate group, the only example is from plant. In crucifer vegetables such as broccoli, cabbage and wasabi, the glucosinolates are hydrolyzed by myrosinase to generate various isothiocyanate-containing compounds [40]. However, no myrosinase homolog-encoding gene exists in the *pau* cluster, implying a different mechanism for the isothiocyanate biosynthesis in paulomycin. The presence of Pau30 (ThiF-like enzyme) and Pau31 (cysteine desulfurase) suggests a cysteine origin of the isothiocyanate sulfur group. Gene *pau31* encodes a protein similar to the cysteine desulfurase IscS, which is a pyridoxal 5ˊ-phosphate dependent enzyme that supplies sulfur by converting L-cysteine to L-alanine and sulfane sulfur [41]. The *pau30* gene encoding a ThiF-like protein transfers the sulfur group released by cysteine desulfurase to the sulfur-carrier protein to form a thiocarboxylate group [42]. We propose that the sulfur group in paulomycin is supplied as in thiamin biosynthesis, in which the thiol group released from cysteine by cysteine desulfurase (IscS) is transferred to the sulfur-carrier protein (ThiS) by ThiF [43]. However, we could not find any ThiS homolog-encoding gene in the *pau* gene cluster, suggesting that a universal sulfur-carrier protein encoded by a gene outside the cluster is recruited in paulomycin biosynthesis, analogous to the 2-thiosugar moiety biosynthesis in BE-7585A [44]. The origins of the isothiocyanate carbon and nitrogen groups and the detailed biosynthetic logic of this unusual moiety are intriguing questions that require further investigation.

### Biosynthesis of the paulomycose moiety

Twelve genes (*pau6*, *pau7*, *pau11*, *pau12*, *pau14-pau16*, *pau22*, *pau23* and *pau42-pau44*) are proposed to be involved in the biosynthesis of the paulomycose moiety. At first, D-glucose-1-phosphate is activated to TDP-D-glucose by Pau23, the putative hexose-1-phosphate thymidylyltransferase. The following steps from TDP-D-glucose to TDP-4″-keto-L-olivose are catalyzed by Pau22 (TDP-hexose 4,6-dehydratase), Pau16 (TDP-hexose 2,3-dehydratase), Pau42 (TDP-4-keto-6-deoxyhexose 2, 3-reductase) and Pau44 (TDP-4-keto-6-deoxyhexose 3, 5-epimerase), in a route parallel to that of deoxysugar biosynthesis in the avermectin biosynthetic pathway [45]. The TDP-6-deoxy-L-hexose 3-O-methyltransferase Pau43 is responsible for the 3″-O-methylation of TDP-4″-keto-L-olivose, and the resulting deoxysugar is then attached to the 3-Oˊ position of the D-allose moiety by Pau14 and Pau15. The gene products of *pau14* and *pau15* show high similarities to cytochrome P450 family protein DesVIII and glycosyltransferase DesVII, both of which are required for attachment of TDP-D-desosamine in pikromycin biosynthesis [46, 47]. The gene products of *pau11* and *pau12* resemble AviB1 (37% identity) and AviB2 (34% identity) from *Streptomyces viridochromogenes* Tü57, respectively. As previously mentioned, AviB1 and AviB2 are α and β subunits of the pyruvate dehydrogenase like proteins involved in the biosynthesis of the two-carbon branched chain sugar methyleurekanate [27]. We propose that the installation of the two-carbon branched chain takes place after the hexose is attached to the D-allose moiety, analogous to methyleurekanate formation in avilamycin biosynthesis, in which the 4-ketosugar precursor of methyleurekanate is attached to the sugar chain before the two-carbon unit is appended [27]. The pyruvate dehydrogenase-like proteins Pau11 and Pau12 hijack a two-carbon unit from pyruvate, which is then loaded onto the C4″ position through an unknown mechanism. Finally, the 7″-keto is reduced to a hydroxyl group by an oxidoreductase (Pau7), and an acyltransferase (Pau6) appends a variety of fatty acids to the 7″-hydroxyl group to form different paulomycins.

### Genes for resistance, regulation and unassigned functions

The *pau9* gene is the only apparent candidate for self-protection within the *pau* gene cluster, which encodes a protein exhibiting 35% identity to a drug resistance transporter AsuM1 from the asukamycin producer *Streptomyces nodosus* subsp. *asukaensis* [48].

There are four putative regulatory genes (*pau4*, *pau5*, *pau13* and *pau32*) in the *pau* cluster. They belong to three transcriptional regulator families including the TetR family (Pau4), the LuxR family (Pau5 and Pau32) and the *S* *treptomyces* antibiotic regulatory protein (SARP) family (Pau13).

There are seven functionally unassigned ORFs in the *pau* cluster including two hypothetical proteins (*pau8* and *pau40*) and five genes with deduced functions that cannot be assigned to paulomycin biosynthesis. The proposed functions of the five remaining genes are elongation factor G (Pau10), glyoxalase (Pau26), enoyl reductase (Pau33), dihydrodipicolinate reductase (Pau36) and pyranose oxidase (Pau41).

### Improving paulomycin production by overexpressing gene pau13

With the paulomycin biosynthetic gene cluster in hand, we set out to increase production of the paulomycins by manipulating its pathway regulation, which has proved to be an efficient strategy in many rational metabolic engineering efforts. The gene product of *pau13* is a SARP family regulator, which is usually functional as an activator stimulating the biosynthesis of antibiotics in *Streptomyces* [3]. The *S*. *paulus pau13*::*aph* mutant CIM3005 was constructed by replacing this gene with a kanamycin resistance cassette. HPLC analysis revealed that production of the paulomycins was almost abolished in CIM3005, suggesting that Pau13 is a positive regulator. The *pau13* mutant complemented strain CIM3006 was constructed by introduction of pCIM3010, in which the *pau13* gene was put under the control of a constitutive promoter ermE*, into CIM3005. When fermented in medium R5α, CIM3006 restored the production of paulomycin A, paulomenol A and paulomenol B, excluding the influence of polar effect. Subsequently, pCIM3010 was introduced into *S*. *paulus* wild-type to generate a *pau13* overexpressing recombination strain CIM3007. The titers of paulomycin A, paulomycin B, paulomenol A and paulomenol B were increased 3.4±0.9, 4.2±1.3, 4.1±0.8 and 4.2±1.2 fold in *S*. *paulus* CIM3006, respectively.

## Conclusions

In summary, we have defined the paulomycin biosynthetic gene cluster by comparative genome mining of three paulomycin producers. A convergent model of paulomycin biosynthesis was proposed after we confirmed the identity of the *pau* gene cluster and determined its boundaries in *S*. *paulus* NRRL 8115. The production of paulomycins was improved significantly by rational engineering of the pathway regulation in *S*. *paulus* NRRL 8115, establishing an excellent foundation for future investigations of paulomycin biosynthesis and engineering.

## Supporting Information

S1 Fig Construction and genotype confirmation of *S*. *paulus* mutants CIM3001, CIM3002 and CIM3005. (A) Diagram illustrating the construction of CIM3001 by replacing *pau11* with a kanamycin-resistance gene (*aph*). (B) PCR detection of *pau11* inactivation. Lane 1, fragments obtained by PCR with CIM3001 as a template and a following *Xba*I digestion; lane 2, fragments obtained by PCR with *S*. *paulus* NRRL 8115 as a template and a following *Xba*I digestion (Expected sizes of PCR fragments after restriction with the indicated enzyme are shown in panel A); Lane 3, DNA Ladder. (C) Diagram illustrating the construction of CIM3002 by replacing *pau18* with a kanamycin-resistance gene. (D) PCR detection of *pau18* inactivation. Lane 1, fragments obtained by PCR with *S*. *paulus* NRRL 8115 as a template; lane 2,fragments obtained by PCR with CIM3002 as a template (Expected sizes of PCR fragments are shown in panel C); Lane 3, DNA Ladder. (E) Diagram illustrating the construction of CIM3005 by replacing *pau13* with a kanamycin-resistance gene. (F) PCR detection of *pau13* inactivation. Lane 1, fragments obtained by PCR with CIM3005 as a template and a following *Bam*HI digestion; lane 2, fragments obtained by PCR with *S*. *paulus* NRRL 8115 as a template and a following *Bam*HI digestion (Expected sizes of PCR fragments after restriction with the indicated enzyme are shown in panel E); Lane 3, DNA Ladder.
(TIF)

S2 Fig Construction and genotype confirmation of *S*. *paulus* mutants CIM3008-CIM3010. (A) Diagram illustrating the construction of CIM3008 by replacing *pau1* with an apramycin-resistance gene (*aac(3)IV*). (B) PCR detection of *pau1* inactivation. Lane 1, fragments obtained by PCR with CIM3008 as a template; lane 2, fragments obtained by PCR with a single-cross mutant as a template; lane 3, fragments obtained by PCR with *S*. *paulus* NRRL 8115 as a template (Expected sizes of PCR fragments are shown in panel A); Lane 4, DNA Ladder. (C) Diagram illustrating the construction of CIM3009 by replacing *pau3* with an apramycin-resistance gene. (D) PCR detection of *pau3* inactivation. Lane 1, fragments obtained by PCR with CIM3009 as a template; lane 2, fragments obtained by PCR with *S*. *paulus* NRRL 8115 as a template (Expected sizes of PCR fragments are shown in panel C); Lane 3, DNA Ladder. (E) Diagram illustrating the construction of CIM3010 by replacing *pau7* with an apramycin-resistance gene. (F) PCR detection of *pau7* inactivation. Lane 1, fragments obtained by PCR with CIM3010 as a template; lane 2, fragments obtained by PCR with *S*. *paulus* NRRL 8115 as a template (Expected sizes of PCR fragments are shown in panel E); Lane 3, DNA Ladder.
(TIF)

S3 Fig Construction and genotype confirmation of *S*. *paulus* mutants CIM3011-CIM3014. (A) Diagram illustrating the construction of CIM3011 by replacing *pau43* with a kanamycin-resistance gene (*aph*). (B) PCR detection of *pau43* inactivation. Lane 1, fragments obtained by PCR with CIM3011 as a template; lane 2, fragments obtained by PCR with *S*. *paulus* NRRL 8115 as a template (Expected sizes of PCR fragments are shown in panel A); Lane 3, DNA Ladder. (C) Diagram illustrating the construction of CIM3012 by replacing *pau45* with an apramycin-resistance gene (*aac(3)IV*). (D) PCR detection of *pau3* inactivation. Lane 1, fragments obtained by PCR with *S*. *paulus* NRRL 8115 as a template and a following *Bln*I digestion; lane 2, fragments obtained by PCR with a single-cross mutant as a template and a following *Bln*I digestion; lane 3, fragments obtained by PCR with CIM3012 as a template and a following *Bln*I digestion (Expected sizes of PCR fragments after restriction with the indicated enzyme are shown in panel C); Lane 4, DNA Ladder. (E) Diagram illustrating the construction of CIM3013 by replacing *pau48* with an apramycin-resistance gene. (F) PCR detection of *pau48* inactivation. Lane 1, fragments obtained by PCR with CIM3013 as a template; lane 2, fragments obtained by PCR with a single-cross mutant as a template; lane 3, fragments obtained by PCR with *S*. *paulus* NRRL 8115 as a template (Expected sizes of PCR fragments are shown in panel E); Lane 4, DNA Ladder. (G) Diagram illustrating the construction of CIM3014 by replacing *pau52* with an apramycin-resistance gene. (H) PCR detection of *pau52* inactivation. Lane 1, fragments obtained by PCR with CIM3014 as a template; lane 2, fragments obtained by PCR with *S*. *paulus* NRRL 8115 as a template (Expected sizes of PCR fragments are shown in panel G); Lane 3, DNA Ladder.
(TIF)

S4 Fig Spectroscopic analyses of paulomycin A. (A) Plausible fragmentation pattern of paulomycin A in tandam MS detection. (B) Tandam MS of paulomycin A. (C) High resolution MS of paulomycin A. (D) UV-vis spectrum of paulomycin A.
(TIF)

S5 Fig Spectroscopic analyses of paulomycin B. (A) Plausible fragmentation pattern of paulomycin B in tandam MS detection. (B) Tandam MS of paulomycin B. (C) High resolution MS of paulomycin B. (D) UV-vis spectrum of paulomycin B.
(TIF)

S6 Fig Spectroscopic analyses of paulomenol A. (A) Plausible fragmentation pattern of paulomenol A in tandam MS detection. (B) Tandam MS of paulomenol A. (C) High resolution MS of paulomenol A. (D) UV-vis spectrum of paulomenol A.
(TIF)

S7 Fig Spectroscopic analyses of paulomenol B. (A) Plausible fragmentation pattern of paulomenol B in tandam MS detection. (B) Tandam MS of paulomenol B. (C) High resolution MS of paulomenol B. (D) UV-vis spectrum of paulomenol B.
(TIF)

S8 Fig HPLC traces of *S*. *paulus* CIM3008-CIM3014 for the *pau* cluster boundaries determination. The inactivated gene of each mutant is bracketed. Paulomycin A (□); Paulomycin B (○); Paulomenol A (⋄); Paulomenol B (Δ).
(TIF)

S9 Fig Multiple alignments of Pau29 and its homologs. The catalytic triad Cys-His-Asp for ketoacylsynthase III-like acyltransferase CerJ is marked with inverted triangles; and the conserved catalytic triad Cys(Ser)-His-His for ketoacylsynthases DpsC and CosE are marked with asterisks. It is notable that the first conserved His in ketoacylsynthases is substituted by Val in CerJ and Asn in Pau29, PauY29 and YP_007743993.
(TIF)

S1 File Construction of the mutants for determination of the paulomycin gene cluster boundaries. (DOC)

S1 Table Strains and plasmids used in this study. (DOC)

S2 Table Primers used in this study. (DOC)
