# Supplementary figures and images for: Identification and Analysis of the Paulomycin Biosynthetic Gene Cluster and Titer Improvement of the Paulomycins in Streptomyces paulus NRRL 8115
Source: PLoS One. 2015 Mar 30;10(3):e0120542. doi: 10.1371/journal.pone.0120542 (PMC4425429; doi:10.1371/journal.pone.0120542)

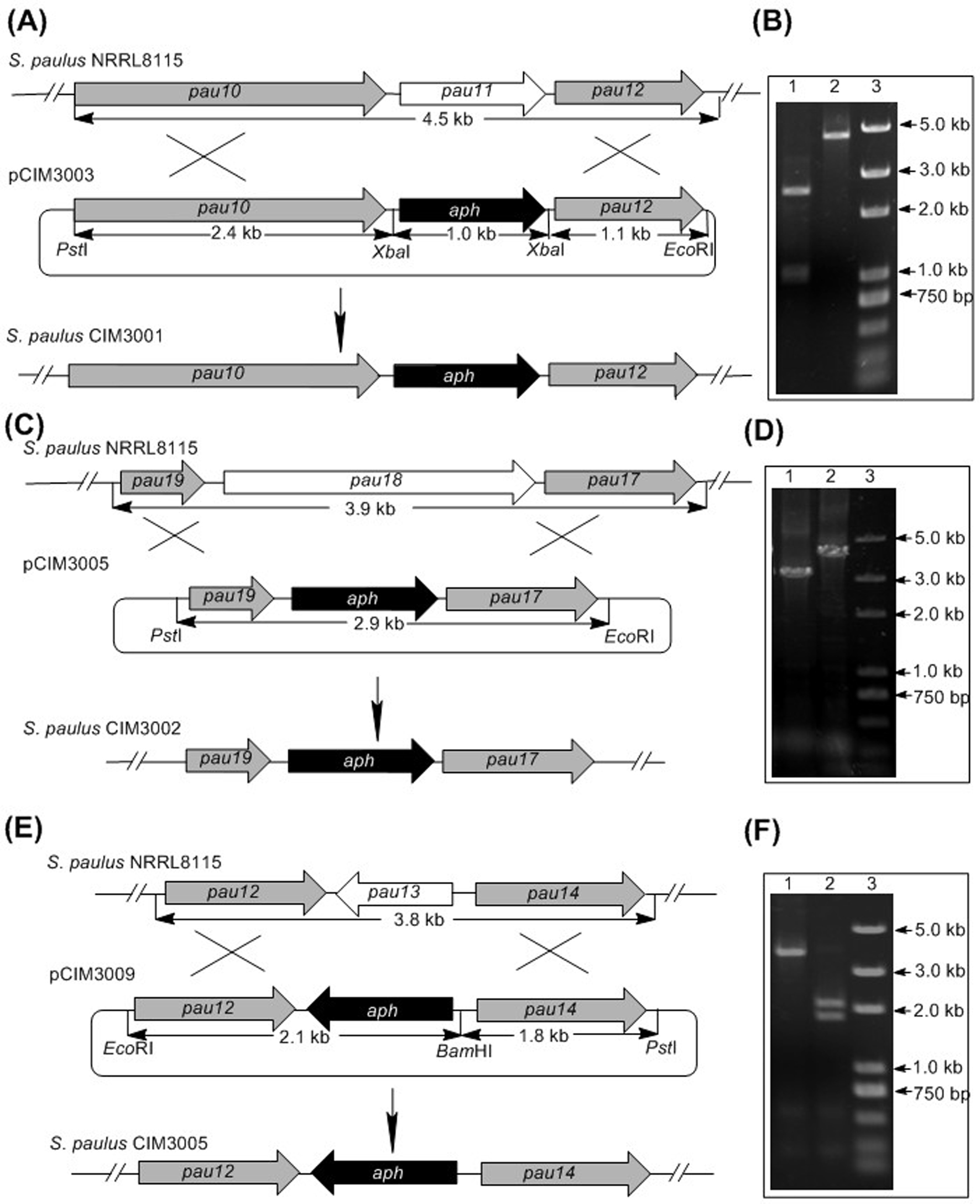

Supplement: S1 Fig — (A) Diagram illustrating the construction of CIM3001 by replacing pau11 with a kanamycin-resistance gene (aph). (B) PCR detection of pau11 inactivation. Lane 1, fragments obtained by PCR with CIM3001 as a template and a following XbaI digestion; lane 2, fragments obtained by PCR with S. paulus NRRL 8115 as a template and a following XbaI digestion (Expected sizes of PCR fragments after restriction with the indicated enzyme are shown in panel A); Lane 3, DNA Ladder. (C) Diagram illustrating the construction of CIM3002 by replacing pau18 with a kanamycin-resistance gene. (D) PCR detection of pau18 inactivation. Lane 1, fragments obtained by PCR with S. paulus NRRL 8115 as a template; lane 2,fragments obtained by PCR with CIM3002 as a template (Expected sizes of PCR fragments are shown in panel C); Lane 3, DNA Ladder. (E) Diagram illustrating the construction of CIM3005 by replacing pau13 with a kanamycin-resistance gene. (F) PCR detection of pau13 inactivation. Lane 1, fragments obtained by PCR with CIM3005 as a template and a following BamHI digestion; lane 2, fragments obtained by PCR with S. paulus NRRL 8115 as a template and a following BamHI digestion (Expected sizes of PCR fragments after restriction with the indicated enzyme are shown in panel E); Lane 3, DNA Ladder. (TIF) [file pone.0120542.s001.tif]

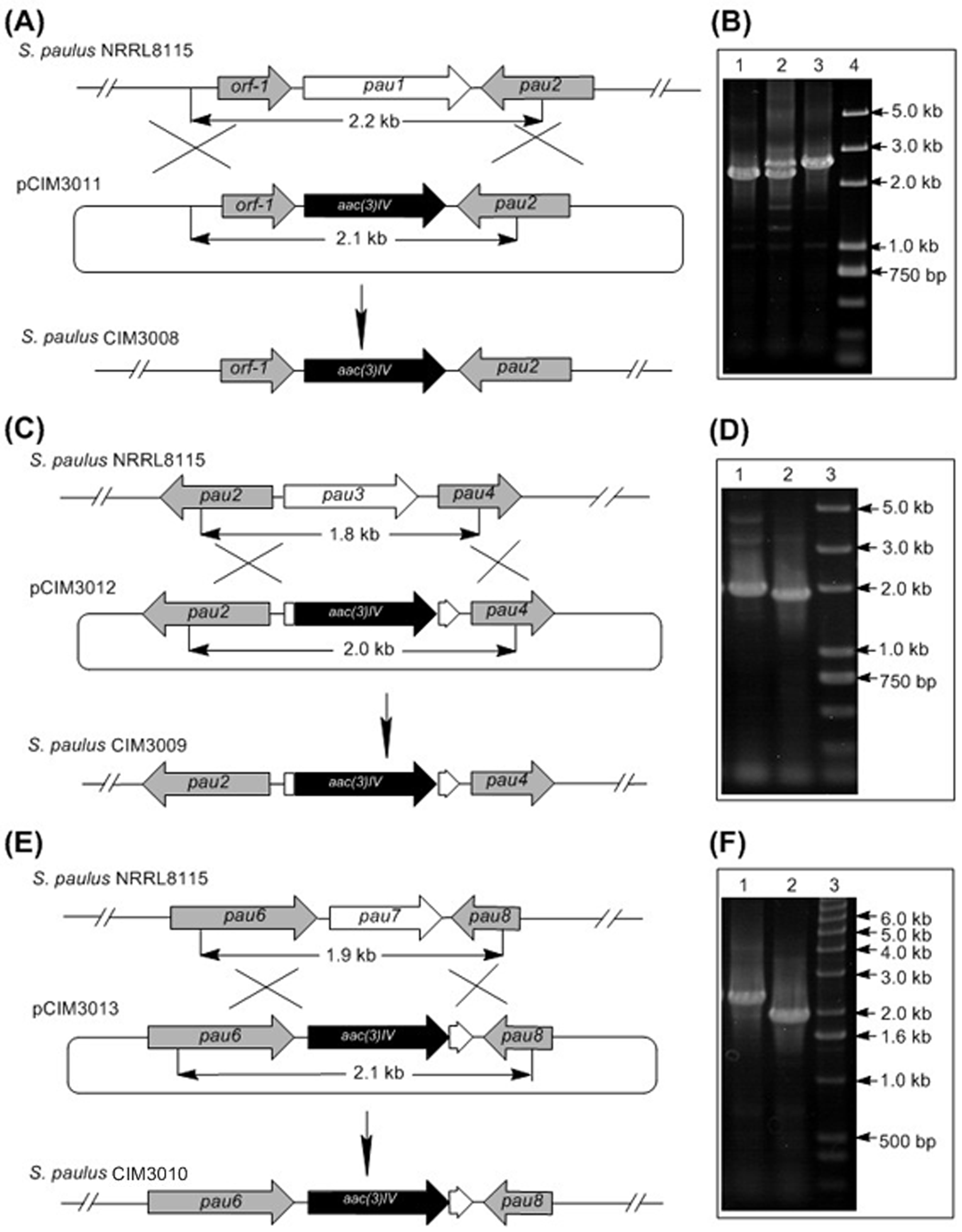

Supplement: S2 Fig — (A) Diagram illustrating the construction of CIM3008 by replacing pau1 with an apramycin-resistance gene (aac(3)IV). (B) PCR detection of pau1 inactivation. Lane 1, fragments obtained by PCR with CIM3008 as a template; lane 2, fragments obtained by PCR with a single-cross mutant as a template; lane 3, fragments obtained by PCR with S. paulus NRRL 8115 as a template (Expected sizes of PCR fragments are shown in panel A); Lane 4, DNA Ladder. (C) Diagram illustrating the construction of CIM3009 by replacing pau3 with an apramycin-resistance gene. (D) PCR detection of pau3 inactivation. Lane 1, fragments obtained by PCR with CIM3009 as a template; lane 2, fragments obtained by PCR with S. paulus NRRL 8115 as a template (Expected sizes of PCR fragments are shown in panel C); Lane 3, DNA Ladder. (E) Diagram illustrating the construction of CIM3010 by replacing pau7 with an apramycin-resistance gene. (F) PCR detection of pau7 inactivation. Lane 1, fragments obtained by PCR with CIM3010 as a template; lane 2, fragments obtained by PCR with S. paulus NRRL 8115 as a template (Expected sizes of PCR fragments are shown in panel E); Lane 3, DNA Ladder. (TIF) [file pone.0120542.s002.tif]

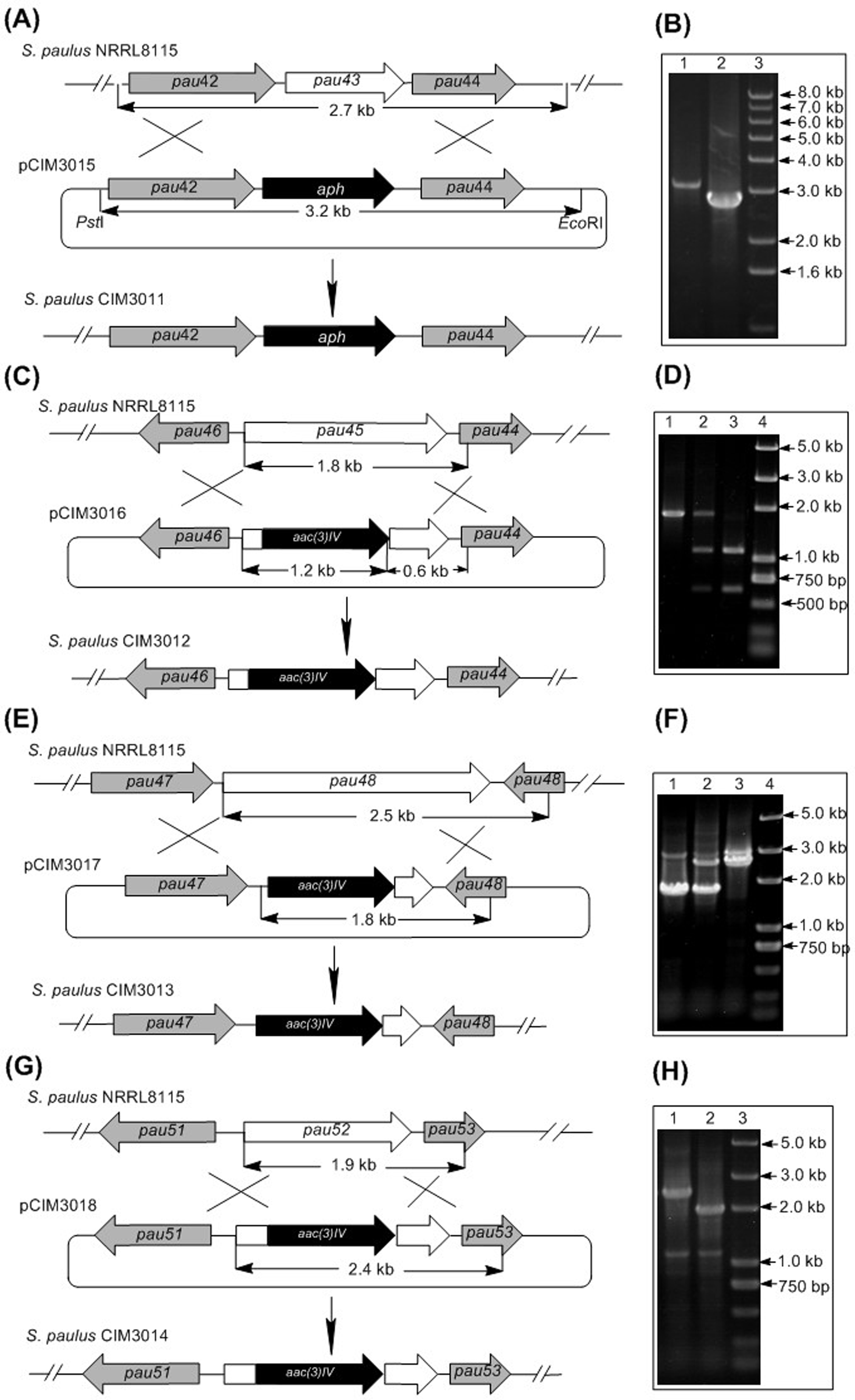

Supplement: S3 Fig — (A) Diagram illustrating the construction of CIM3011 by replacing pau43 with a kanamycin-resistance gene (aph). (B) PCR detection of pau43 inactivation. Lane 1, fragments obtained by PCR with CIM3011 as a template; lane 2, fragments obtained by PCR with S. paulus NRRL 8115 as a template (Expected sizes of PCR fragments are shown in panel A); Lane 3, DNA Ladder. (C) Diagram illustrating the construction of CIM3012 by replacing pau45 with an apramycin-resistance gene (aac(3)IV). (D) PCR detection of pau3 inactivation. Lane 1, fragments obtained by PCR with S. paulus NRRL 8115 as a template and a following BlnI digestion; lane 2, fragments obtained by PCR with a single-cross mutant as a template and a following BlnI digestion; lane 3, fragments obtained by PCR with CIM3012 as a template and a following BlnI digestion (Expected sizes of PCR fragments after restriction with the indicated enzyme are shown in panel C); Lane 4, DNA Ladder. (E) Diagram illustrating the construction of CIM3013 by replacing pau48 with an apramycin-resistance gene. (F) PCR detection of pau48 inactivation. Lane 1, fragments obtained by PCR with CIM3013 as a template; lane 2, fragments obtained by PCR with a single-cross mutant as a template; lane 3, fragments obtained by PCR with S. paulus NRRL 8115 as a template (Expected sizes of PCR fragments are shown in panel E); Lane 4, DNA Ladder. (G) Diagram illustrating the construction of CIM3014 by replacing pau52 with an apramycin-resistance gene. (H) PCR detection of pau52 inactivation. Lane 1, fragments obtained by PCR with CIM3014 as a template; lane 2, fragments obtained by PCR with S. paulus NRRL 8115 as a template (Expected sizes of PCR fragments are shown in panel G); Lane 3, DNA Ladder. (TIF) [file pone.0120542.s003.tif]

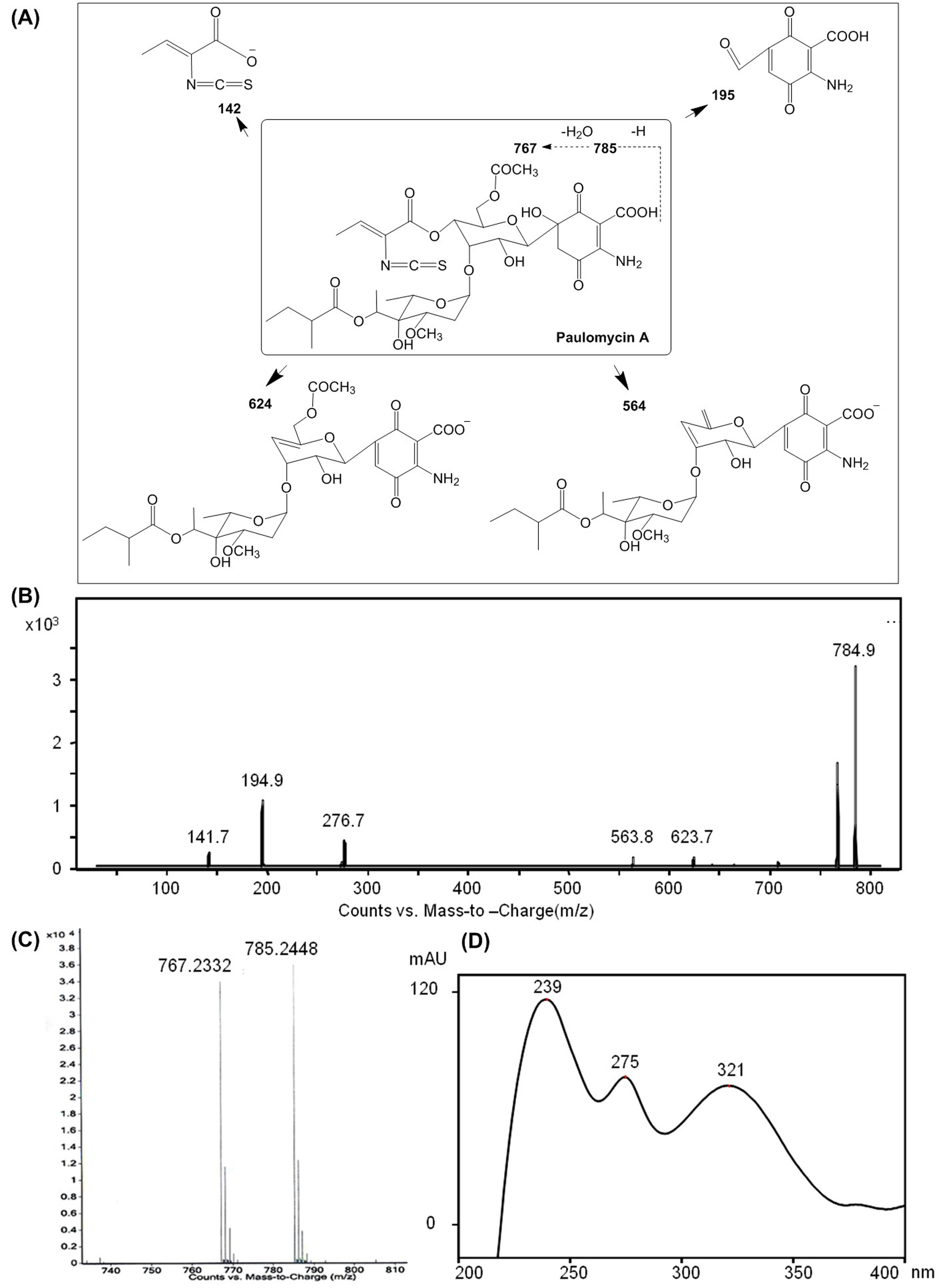

Supplement: S4 Fig — (A) Plausible fragmentation pattern of paulomycin A in tandam MS detection. (B) Tandam MS of paulomycin A. (C) High resolution MS of paulomycin A. (D) UV-vis spectrum of paulomycin A. (TIF) [file pone.0120542.s004.tif]

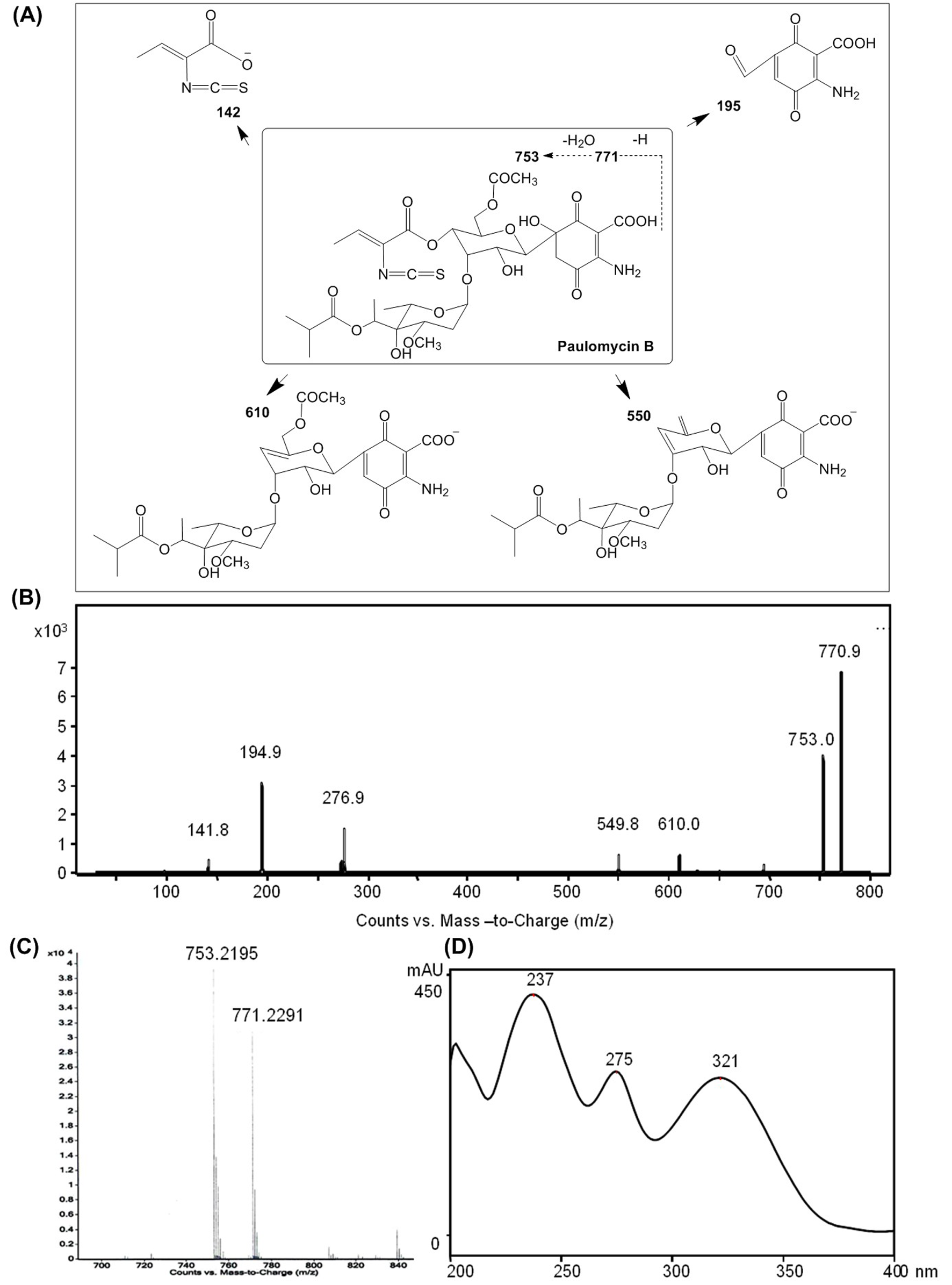

Supplement: S5 Fig — (A) Plausible fragmentation pattern of paulomycin B in tandam MS detection. (B) Tandam MS of paulomycin B. (C) High resolution MS of paulomycin B. (D) UV-vis spectrum of paulomycin B. (TIF) [file pone.0120542.s005.tif]

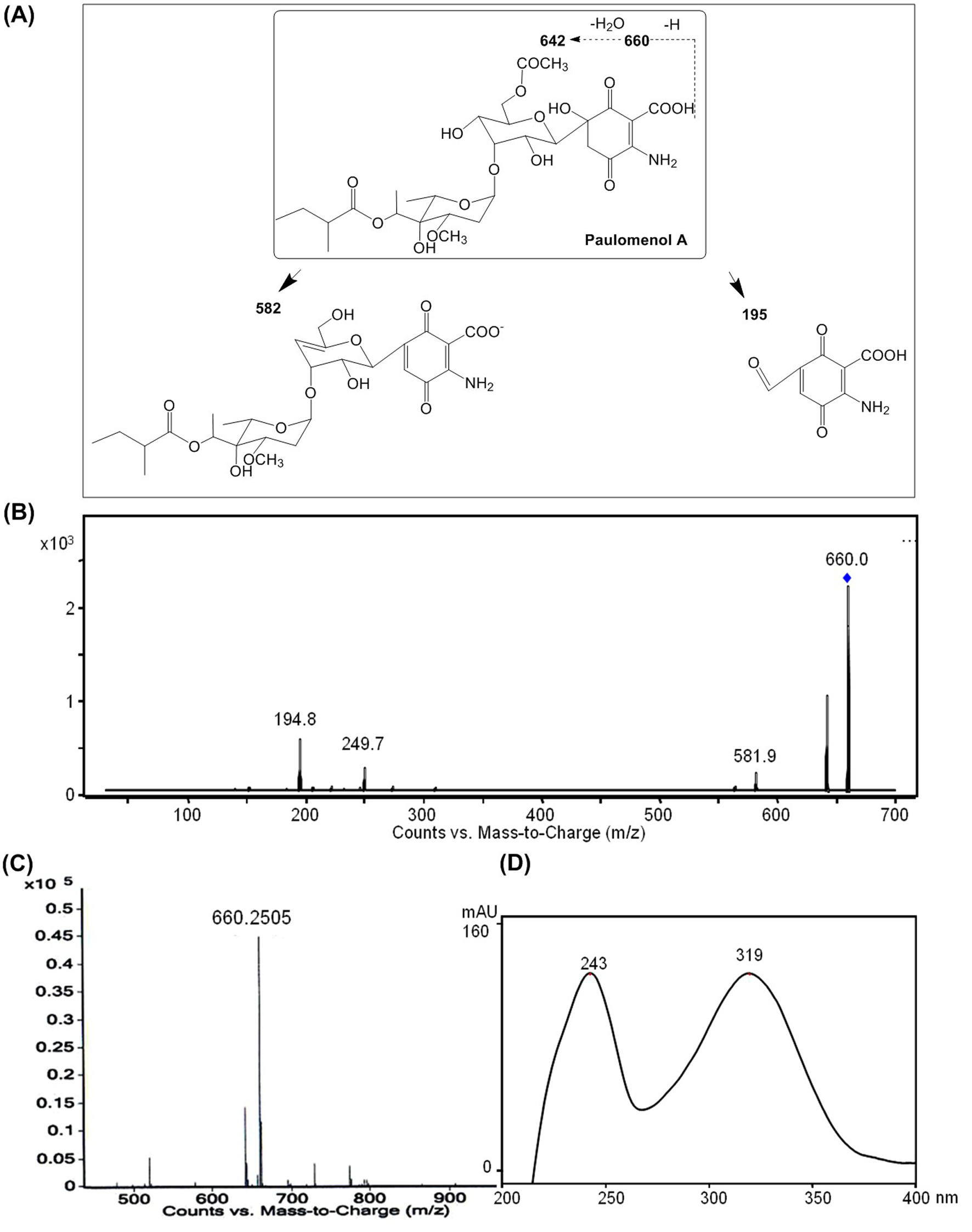

Supplement: S6 Fig — (A) Plausible fragmentation pattern of paulomenol A in tandam MS detection. (B) Tandam MS of paulomenol A. (C) High resolution MS of paulomenol A. (D) UV-vis spectrum of paulomenol A. (TIF) [file pone.0120542.s006.tif]

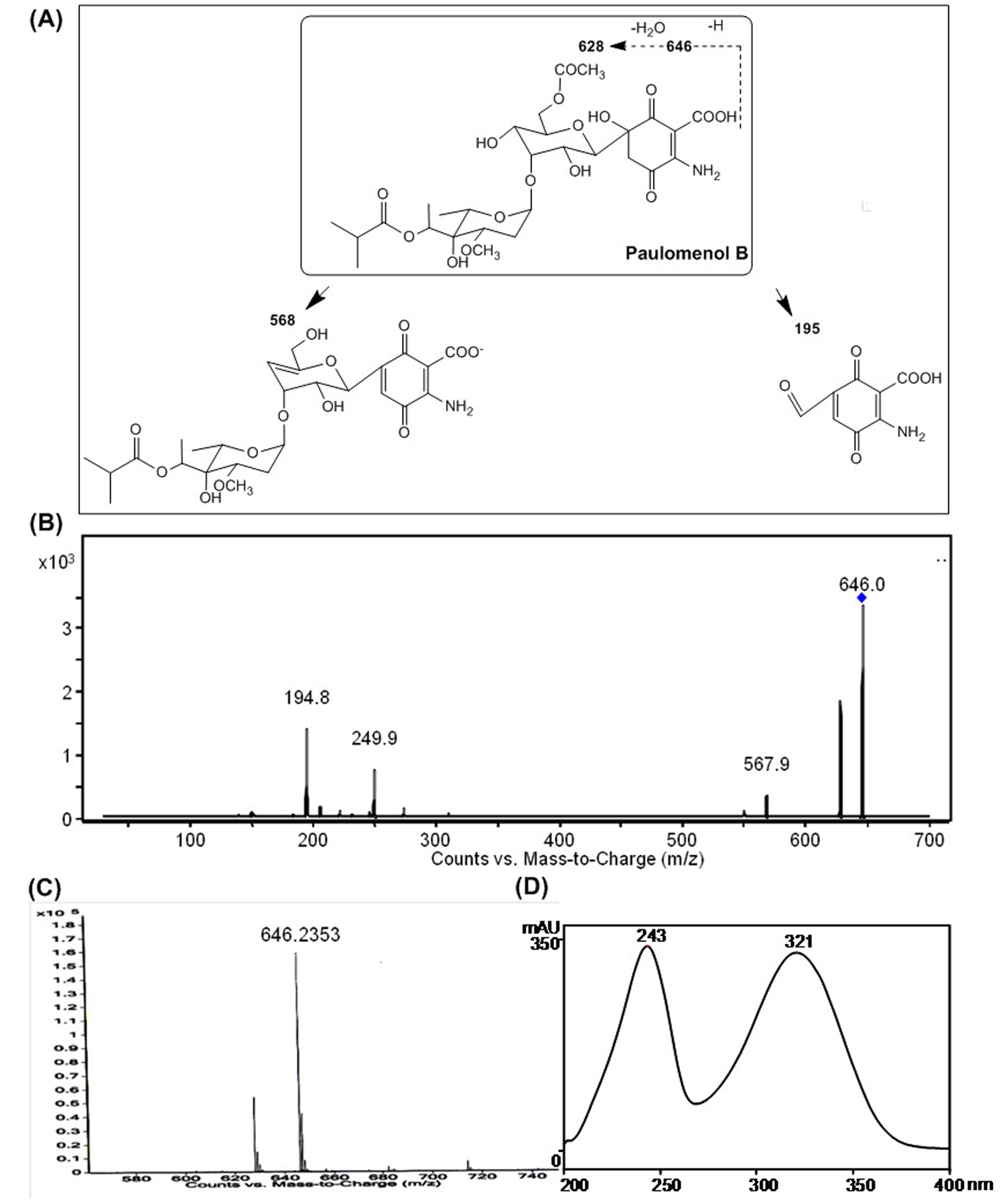

Supplement: S7 Fig — (A) Plausible fragmentation pattern of paulomenol B in tandam MS detection. (B) Tandam MS of paulomenol B. (C) High resolution MS of paulomenol B. (D) UV-vis spectrum of paulomenol B. (TIF) [file pone.0120542.s007.tif]

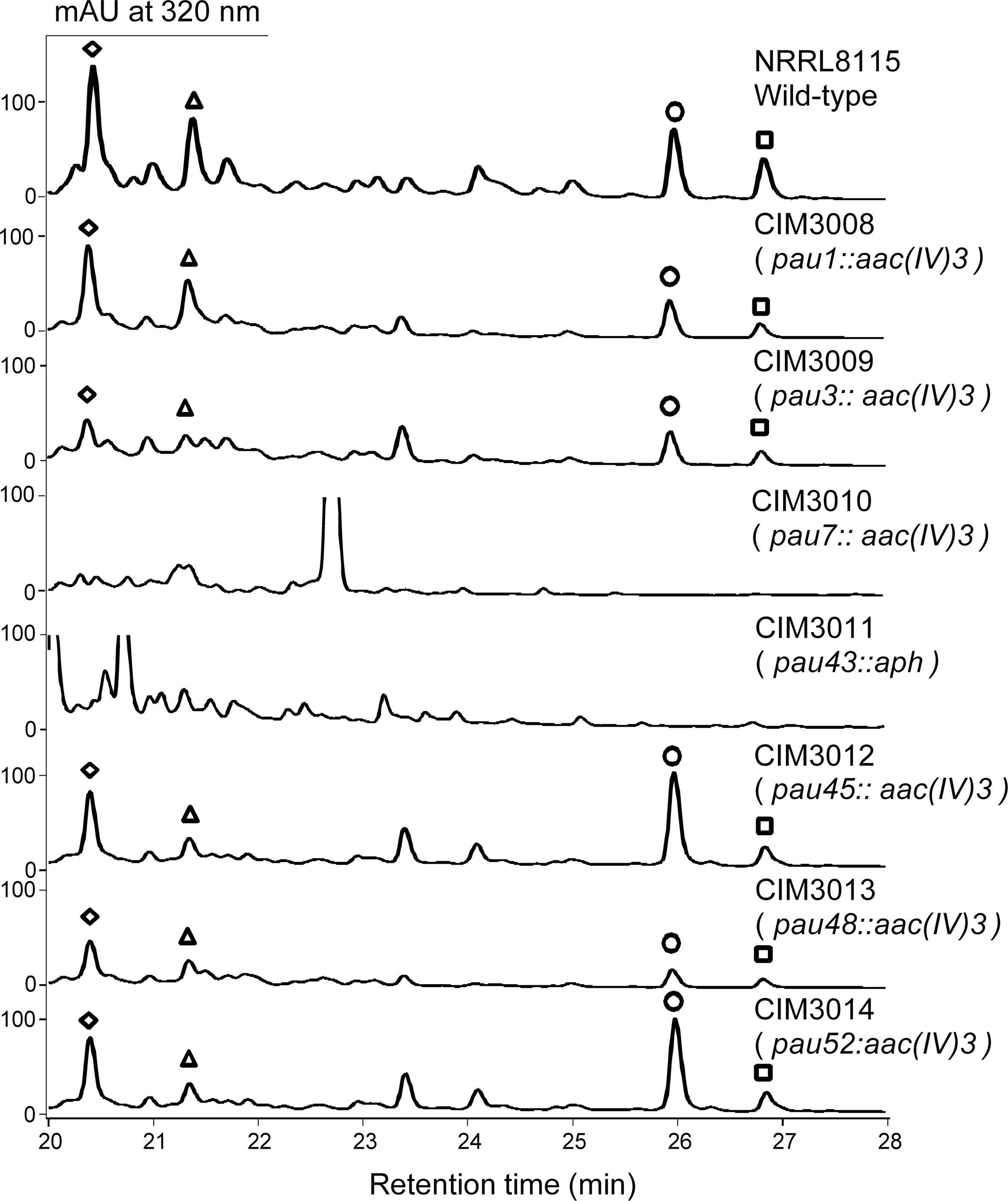

Supplement: S8 Fig — The inactivated gene of each mutant is bracketed. Paulomycin A (□); Paulomycin B (○); Paulomenol A (⋄); Paulomenol B (Δ). (TIF) [file pone.0120542.s008.tif]

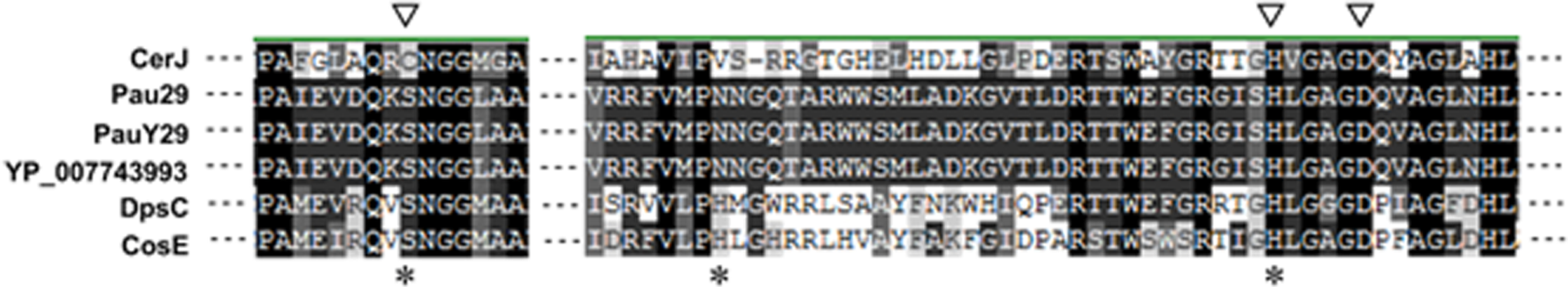

Supplement: S9 Fig — The catalytic triad Cys-His-Asp for ketoacylsynthase III-like acyltransferase CerJ is marked with inverted triangles; and the conserved catalytic triad Cys(Ser)-His-His for ketoacylsynthases DpsC and CosE are marked with asterisks. It is notable that the first conserved His in ketoacylsynthases is substituted by Val in CerJ and Asn in Pau29, PauY29 and YP_007743993. (TIF) [file pone.0120542.s009.tif]
