# Supplementary material for: Identification and Analysis of the Paulomycin Biosynthetic Gene Cluster and Titer Improvement of the Paulomycins in Streptomyces paulus NRRL 8115
Source: PLoS One. 2015 Mar 30;10(3):e0120542. doi: 10.1371/journal.pone.0120542 (PMC4425429; doi:10.1371/journal.pone.0120542)
Supplement: S1 File — (DOC) [file pone.0120542.s010.doc]

## Construction of the mutants for determination of the paulomycin gene cluster boundaries

To determine the bondaries of the paulomycin biosynthetic gene cluster, genes *pau1*, *pau3*, *pau7*, *pau43*, *pau45*, *pau48* and *pau52* were inactivated using the allelic replacement strategy.

### Construction of the CIM3008 (*S. paulus pau1::aac(3)IV*) mutant

The two fragments flanking *pau1* were amplified by PCR using primer pair pau1-up-F and pau1-up-R for the 1.5-kb upstream fragment and primer pair pau1-down-Fand pau1- down -R for the 1.3-kb downstream fragment. The two fragments were inserted into the *Bln*I and *Mun*I sites of pCIMt002 respectively *via* LIC strategy to generate pCIM3011. Introduction of plasmid pCIM3011 into *S. paulus* NRRL 8115 was carried out by *E. coli-Streptomyces* conjugation. Exconjugants with apramycin resistance and without blue pigment were selected as the desired *S. paulus pau1::aac(3)IV* mutant strain CIM3008. The genotype of CIM3008 was confirmed by PCR with primers pau2-down-F and pau2-down-R and subsequent *Xba*I digestion (Figure S2A, S2B).

### Construction of the CIM3009 (*S. paulus pau3::aac(3)IV*) mutant

The two fragments flanking *pau3* were amplified by PCR using primer pair pau3-up-F and pau3-up-R for the 1.3-kb upstream fragment and primer pair pau3-down-F and pau3-down-R for the 1.4-kb downstream fragment. The two fragments were inserted into the *Bln*I and *Mun*I sites of pCIMt002 respectively *via* LIC strategy to generate pCIM3012. Introduction of plasmid pCIM3012 into *S. paulus* NRRL 8115 was performed by *E. coli-Streptomyces* conjugation. Exconjugants with apramycin resistance and without blue pigment were selected as the desired *S. paulus pau3::aac(3)IV* mutant strain CIM3009. The genotype of CIM3009 was confirmed by PCR with primers pau2-up-F and pau2-up-R (Figure S2C, S2D).

### Construction of the CIM3010 (*S. paulus pau7::aac(3)IV*) mutant

The two fragments flanking *pau7* were amplified by PCR using primer pair pau7-up-F and pau7-up-R for the 2.1-kb upstream fragment and primer pair pau7-down-F and pau7-down-R for the 1.8-kb downstream fragment. The two fragments were inserted into the *Bln*I and *Mun*I sites of pCIMt002 respectively *via* LIC strategy to generate pCIM3013. Introduction of plasmid pCIM3013 into *S. paulus* NRRL 8115 was carried out by *E. coli-Streptomyces* conjugation. Exconjugants with apramycin resistance and without blue pigment were selected as the desired *S. paulus pau7::aac(3)IV* mutant strain CIM3010. The genotype of CIM3010 was confirmed by PCR with primers pau6-down-F and pau6-down-R (Figure S2E, S2F).

### Construction of the CIM3011 (*S. paulus pau43::aph*) mutant

The 1.1-kb upstream framgent of *pau43* were amplified using primer pairs pau43-up-F and pau43-up-R. The 1.1-kb downsteam fragment of *pau43* were amplified using primer pairs pau43-down-F and pau43-down-R. Then the two fragments were inserted into *Pst*I*/Bam*HI and *Kpn*I/*Bam*HI sites of pUC119::KanR respectively to generate pCIM3014. The 3.2-kb mutant allele containing the up- and downstream-fragments of *pau43* and the kanamycin resistance cassette was excised by *Pst*I/*Eco*RI and inserted into the same sites of pKC1132 to afford pCIM3015. Plasmid pCIM3015 was then introduced into *S. paulus* NRRL 8115 via *E. coli-Streptomyces* conjugation. Exconjugants with kanamycin resistance and apramycin sensitivity were selected as the desired *S. paulus pau43::aph* mutant strain CIM3011. The genotype of CIM3011 was confirmed by PCR with primers pau43-up-F and pau43-down-R (Figure S3A, S3B).

### Construction of the CIM3012 (*S. paulus pau45::aac(3)IV*) mutant

The two fragments flanking *pau45* were amplified by PCR using primer pair pau45-up-F and pau45-up-R for the 1.5-kb upstream fragment and primer pair pau45-down-F and pau45-down-R for the 1.8-kb downstream fragment. The two fragments were inserted into the *Bln*I and *Mun*I sites of pCIMt002 respectively *via* LIC strategy to generate pCIM3016. Introduction of plasmid pCIM3016 into *S. paulus* NRRL 8115 was carried out via *E. coli-Streptomyces* conjugation. Exconjugants with apramycin resistance and without blue pigment were selected as the desired *S. paulus pau45::aac(3)IV* mutant strain CIM3012. The genotype of CIM3012 was confirmed by PCR with primers pau45-E-F and pau45-E-R (Figure S3C, S3D).

### Construction of the CIM3013 (*S. paulus pau48::aac(3)IV*) mutant

The two fragments flanking *pau48* were amplified by PCR using primer pair pau48-up-F and pau48-up-R for the 1.5-kb upstream fragment and primer pair pau48-down-F and pau48-down-R for the 2.0-kb downstream fragment. The two fragments were inserted into the *Bln*I and *Mun*I sites of pCIMt002 respectively *via* LIC strategy to generate pCIM3017. Introduction of plasmid pCIM3017 into *S. paulus* NRRL 8115 via *E. coli-Streptomyces* conjugation was carried out. Exconjugants with apramycin resistance and without blue pigment were selected as the desired *S. paulus pau48::aac(3)IV* mutant strain CIM3013. The genotype of CIM3013 was confirmed by PCR with primers pau48-E-F and pau48-E-R (Figure S3E, S3F).

### Construction of the CIM3014 (*S. paulus pau52::aac(3)IV*) mutant

The two fragments flanking *pau52* were amplified by PCR using primer pair pau52-up-F and pau52-up-R for the 1.7-kb upstream fragment and primer pair pau52-down-F (MunI) and pau52-down-R for the 1.4-kb downstream fragment. The two fragments were inserted into the *Bln*I and *Mun*I sites of pCIMt002 respectively *via* LIC strategy to generate pCIM3018. Introduction of plasmid pCIM3018 into *S. paulus* NRRL 8115 via *E. coli-Streptomyces* conjugation was carried out. Exconjugants with apramycin resistance and without blue pigment were selected as the desired *S. paulus pau52::aac(3)IV* mutant strain CIM3014. The genotype of CIM3014 was confirmed by PCR with primers Pau52-E-F and Pau52-E-R (Figure S3G, S3H).
