# Supplementary material for: Identification and Analysis of the Paulomycin Biosynthetic Gene Cluster and Titer Improvement of the Paulomycins in Streptomyces paulus NRRL 8115
Source: PLoS One. 2015 Mar 30;10(3):e0120542. doi: 10.1371/journal.pone.0120542 (PMC4425429; doi:10.1371/journal.pone.0120542)
Supplement: S1 Table — (DOC) [file pone.0120542.s011.doc]

**Table S1. Strains and plasmids used in this study**

| **Strains** | **Descriptions** | **References** |
| --- | --- | --- |
| *E. coli* JM109 | *E. coli* host for cloning | Commercial |
| *E. coli* ET12567/pUZ8002 | *E. coli* host for conjugation | *Ref.* 19 |
| *S. paulus* NRRL 8115 | Wild type paulomycin producer | *Ref.* 6 |
| *S. paulus* CIM3001 | NRRL8115 *pau11*::*aph* | This study |
| *S. paulus* CIM3002 | NRRL8115 *pau18*::*aph* | This study |
| *S. paulus* CIM3003 | *S. paulus* CIM3001 harboring pCIM3006 | This study |
| *S. paulus* CIM3004 | *S. paulus* CIM3002 harboring pCIM3007 | This study |
| *S. paulus* CIM3005 | NRRL8115 *pau13*::*aph* | This study |
| *S. paulus* CIM3006 | *S. paulus* CIM3005 harboring pCIM3010 | This study |
| *S. paulus* CIM3007 | NRRL8115 harboring pCIM3010 | This study |
| *S. paulus* CIM3008 | NRRL8115 *pau1*::*aac(IV)3* | This study |
| *S. paulus* CIM3009 | NRRL8115 *pau3*:: *aac(IV)3* | This study |
| *S. paulus* CIM3010 | NRRL8115 *pau7*:: *aac(IV)3* | This study |
| *S. paulus* CIM3011 | NRRL8115 *pau43*::*aph* | This study |
| *S. paulus* CIM3012 | NRRL8115 *pau45*:: *aac(IV)3* | This study |
| *S. paulus* CIM3013 | NRRL8115 *pau48*::*aac(IV)3* | This study |
| *S. paulus* CIM3014 | NRRL8115 *pau52*:*aac(IV)3* | This study |
| **Plasmids** | **Descriptions** | **References** |
| pUC119::KanR | Contains kanamycin resistance cassette (*aph*) | *Ref.* 21 |
| pKC1132 | *E. coli-Streptomyces* shuttle vector | *Ref.* 19 |
| pCIMt002 | *E. coli-Streptomyces* shuttle vector, with indigodine synthetase gene | In press |
| pSET152::ermE* | *E. coli-Streptomyces* shuttle vector, with *ermE** promoter | *Ref.* 22 |
| pCIM3001 | pUC119 *pau11-up*::*aph*, generated by inserted the upstream fragment into the *Pst*I*/Xba*I sites of pUC119::aph | This study |
| pCIM3002 | pUC119 *pau11*::*aph*, generated by inserted the downstream fragment into the *Eco*RI site of pCIM3001 | This study |
| pCIM3003 | pKC1132 *pau11*::*aph*, generated by inserted the *pau11*::*aph* mutant allele into the *Pst*I/*Eco*RI sites of pKC1132 | This study |
| pCIM3004 | pUC119 *pau18*::*aph*, generated by inserted the up- and downstream fragments of *pau18* into the *Pst*I/*Bam*HI and *Kpn*I/*Eco*RI sites of pUC119::KanR respectively | This study |
| pCIM3005 | pKC1132 *pau18*::*aph*, generated by inserted the *pau18*::*aph* mutant allele into the *Pst*I/*Eco*RI sites of pKC1132 | This study |
| pCIM3006 | pSET152 derivative harboring the *pau11* gene under control of promoter *ermE** | This study |
| pCIM3007 | pSET152 derivative harboring the *pau18* gene under control of promoter *ermE** | This study |
| pCIM3008 | pUC119 *pau13*::*aph*, generated by inserted the up- and downstream fragments of *pau13* into the *Eco*RI/*Kpn*I and *BamH*I/*Pst*I sites of pUC119::KanR respectively | This study |
| pCIM3009 | pKC1132 *pau13*::*aph*, generated by inserted the *pau13*::*aph* mutant allele into the *Eco*RI/*Pst*I sites of pKC1132 | This study |
| pCIM3010 | pSET152 derivative harboring the *pau13* gene under control of promoter *ermE** | This study |
| pCIM3011 | pCIMt002 *pau1*::*aac(IV)3,* generated by inserted the up- and downstream fragments of *pau1* into the *Bln*I and *Eco*RI sites of pCIMt002 respectively | This study |
| pCIM3012 | pCIMt002 *pau3*::*aac(IV)3,* generated by inserted the up- and downstream fragments of *pau3* into the *Bln*I and *Eco*RI sites of pCIMt002 | This study |
| pCIM3013 | pCIMt002 *pau7*::*aac(IV)3,* generated by inserted the up- and downstream fragments of *pau7* into the *Bln*I and *Eco*RI sites of pCIMt002 | This study |
| pCIM3014 | pUC119 *pau43*::*aph*, generated by inserted the up- and downstream fragments of *pau43* into the *Pst*I/*Bam*HI and *Kpn*I/*Eco*RI sites of pUC119::KanR respectively | This study |
| pCIM3015 | pKC1132 *pau43*::*aph*, generated by inserted the *pau43*::*aph* mutant allele into the *Pst*I/*Eco*RI sites of pKC1132 | This study |
| pCIM3016 | pCIMt002 *pau45*::*aac(IV)3,* generated by inserted the up- and downstream fragments of *pau45* into the *Bln*I and *Eco*RI sites of pCIMt002 | This study |
| pCIM3017 | pCIMt002 *pau48*::*aac(IV)3,* generated by inserted the up- and downstream fragments of *pau48* into the *Bln*I and *Eco*RI sites of pCIMt002 | This study |
| pCIM3018 | pCIMt002 *pau52*::*aac(IV)3,* generated by inserted the up- and downstream fragments of *pau52* into the *Bln*I and *Eco*RI sites of pCIMt002 | This study |
