# Supplementary material for: Identification and Analysis of the Paulomycin Biosynthetic Gene Cluster and Titer Improvement of the Paulomycins in Streptomyces paulus NRRL 8115
Source: PLoS One. 2015 Mar 30;10(3):e0120542. doi: 10.1371/journal.pone.0120542 (PMC4425429; doi:10.1371/journal.pone.0120542)
Supplement: S2 Table — (DOC) [file pone.0120542.s012.doc]

**Table S2. Primers used in this study**

| **Name** | **Sequence (5'-3')** | **Restriction enzyme sites** |
| --- | --- | --- |
| pau11-up-F | AAAACTGCAGCATATGGCTACCACTTCGCTCGATC | *Pst*I |
| pau11-up-R | GCTCTAGACGACGACACGGATACGCACC | *Xba*I |
| pau11-down-F | TTCGGGTACCGAGCTCGAATTTCTAGACACGGATAAGGAGCACAGC | *Xba*I |
| pau11-down-R | TAAAACGACGGCCAGTGAATTCGCGAGGGAAGGGGAAGGCTA | *Eco*RI |
| pau11-E-F | AAAACTGCAGCATATGGTTTCTGATCGTGCACGACTG | *Nde*I |
| pau11-E-R | CGGGATCCTGCTGTGCTCCTTATCCGTGGG | *Bam*HI |
| pau13-up-F | GGAATTCCATATGCGCACACTGACGTACAGGC | *Eco*RI |
| pau13-up-R | GGGGTACCGCGAGGGAAGGGGAAGGCTA | *Kpn*I |
| pau13-down-F | CGGGATCCGCAGTTGGCGAGGCTTGTTC | *Bam*HI |
| pau13-down-R | AAAACTGCAGATCGGATGGAAGTGCGTGGG | *Pst*I |
| pau13-E-F | TAAAACGACGGCCAGTGAATTCCATATGCAGGCCGAGAACAAGACAGC | *Nde*I |
| pau13-E-R | CGGGATCCCCCTTCCCTCGCACGGCTC | *Bam*HI |
| pau18-up-F | AAAACTGCAGCATATGGCCATGCCCAGTATCGCTC | *Pst*I |
| pau18-up-R | CGGGATCCGAGCGGTTTCAGGTACGGGTGC | *Bam*HI |
| pau18-down-F | GGGGTACCTCTAGAGCTGAGAATCGTCCGCTGCAC | *Kpn*I |
| pau18-down-R | GGAATTCGTTCGCTGGTCACCTGCCTC | *Eco*RI |
| pau18-E-F | AAAACTGCAGCATATGACCACCGACCAGGCCCG | *Nde*I |
| pau18-E-R | CGGGATCCGCCGCCTGCGATGACGAAC | *Bam*HI |
| pau1-up-F | CTTCCATGGGCACGCCCTAGGGTCCTGCTGCCGTTCTTCTCC | *Bln*I |
| pau1-up-R | ATCCCTTAACGTGAGCCTAGGCGGTGATCAGCCAGCCCTTG | *Bln*I |
| pau1-down-F | CAGTCGATTGGCTGACAATTGGCCGCAGATCGTCGAGTGGT | *Mun*I |
| pau1-down-R | CTTGCTAGCAGATGTCAATTGGCTCAGCATCTTCCAGACCCTCA | *Mun*I |
| pau2-up-F | CTTCCATGGGCACGCCCTAGGCCACCCTGGTCCGTATCTCCTG | *Bln*I |
| pau2-up-R | ATCCCTTAACGTGAGCCTAGGGCCGTGGTCGATGGAGATGTAGT | *Bln*I |
| pau2-down-F | CAGTCGATTGGCTGACAATTGAACGCCGAGGGCTACACCAAC | *Mun*I |
| pau2-down-R | CTTGCTAGCAGATGTCAATTGCGCTCTTCATCGGCTTCCACC | *Mun*I |
| pau3-up-F | CTTCCATGGGCACGCCCTAGGGCCGCAGATCGTCGAGTGGT | *Bln*I |
| pau3-up-R | ATCCCTTAACGTGAGCCTAGGGCTCAGCATCTTCCAGACCCTCA | *Bln*I |
| pau3-down-F | CAGTCGATTGGCTGACAATTGTACCGGATCTGCACGGCAAG | *Mun*I |
| pau3-down-R | CTTGCTAGCAGATGTCAATTGTATTTCGTCTGGTGTCCCAAGGTC | *Mun*I |
| pau6-up-F | CTTCCATGGGCACGCCCTAGGAGCACCTTTCACGGGTACATCAG | *Bln*I |
| pau6-up-R | ATCCCTTAACGTGAGCCTAGGGGCGACCGTGGCCTTTGTAC | *Bln*I |
| pau6-down-F | CAGTCGATTGGCTGACAATTGGGTGCGGCTGGGAGACATCA | *Mun*I |
| pau6-down-R | CTTGCTAGCAGATGTCAATTGGATCCGCTCATCGTTCGTCAA | *Mun*I |
| pau7-upF | CTTCCATGGGCACGCCCTAGGCTTGGGACACCAGACGAAATACC | *Bln*I |
| pau7-up-R | ATCCCTTAACGTGAGCCTAGGGTGTTGAGGGTGACGGAGGGA | *Bln*I |
| pau7-down-F | CAGTCGATTGGCTGACAATTGGCCCGCAAGCATGGAAAGAG | *Mun*I |
| pau7-down-R | CTTGCTAGCAGATGTCAATTGGCACGGACAGCACGATGACG | *Mun*I |
| pau43-up-F | AAAACTGCAGCATATGTGTCGTCCGATGCAGCG | *Pst*I |
| pau43-up-R | CGGGATCCGGCCATCAGGGCTCCATTCC | *Bam*HI |
| pau43-down-F | TTCGGGTACCGAGCTCGAATCCGCCTACTTCGACCTGGACCTCT | *Kpn*I |
| pau43-down-R | TAAAACGACGGCCAGTGAATTCCCTGCTGCTGACCGTCTCCCT | *Eco*RI |
| pau45-up-F | CTTCCATGGGCACGCCCTAGGCGCTTGTTGCAGGGCATGGAG | *Bln*I |
| pau45-up-R | ATCCCTTAACGTGAGCCTAGGGGGGGGTGAAGCGGTGGTG | *Bln*I |
| pau45-down-F | CAGTCGATTGGCTGACAATTGCGAGAAGCCGAACCAGAAGG | *Mun*I |
| pau45-down-R | CTTGCTAGCAGATGTCAATTGGGCGTCATCATGGAGTTCGG | *Mun*I |
| pau45-E-F | AAAACTGCAGCATATGTCCGCACCCGCGTCCCC | *Nde*I |
| pau45-E-R | CGGGATCCGACTGCTGCCGGAGTACGAGACCTG | *Bam*HI |
| pau48-up-F | CTTCCATGGGCACGCCCTAGGGGTACGAGCTGAGTCTCGACAACC | *Bln*I |
| pau48-up-R | ATCCCTTAACGTGAGCCTAGGGTTCCGAGGTGGTGTCGGTCAT | *Bln*I |
| pau48-down-F | CAGTCGATTGGCTGACAATTGAGTTCCTCGGCGGCATGGTGAT | *Mun*I |
| pau48-down-R | CTTGCTAGCAGATGTCAATTGCCAAGCACTCGATGCGGATGTTC | *Mun*I |
| pau48-E-F | AAAACTGCAGCATATGACCGACACCACCTCGGAAC | *Nde*I |
| pau48-E-R | CGGGATCCGCTGCGTCACCGTGGAGAACC | *Bam*HI |
| pau52-up-F | CTTCCATGGGCACGCCCTAGGATGGACAGGGGACAGCAGGAG | *Bln*I |
| pau52-up-R | ATCCCTTAACGTGAGCCTAGGCGGTGTCCTGCTTGACCTTGA | *Bln*I |
| pau52-down-F | CAGTCGATTGGCTGACAATTGTCAAGTGCTGCCCGCCCATC | *Mun*I |
| pau52-down-R | CTTGCTAGCAGATGTCAATTGAGTCGCCGGTTTCCTGTTGCTC | *Mun*I |
| Pau52-E-F | AAAACTGCAGCATATGCCGGAACTGGAACGGGTAC | *Nde*I |
| Pau52-E-R | CGGGATCCGCGTAGTGGTCGGCGGTGTA | *Bam*HI |
